# Current practices of physiotherapists in Switzerland regarding fall risk-assessment for community-dwelling older adults: A national cross-sectional survey

**DOI:** 10.12688/f1000research.73636.2

**Published:** 2023-12-11

**Authors:** Morgane Duc, Anne-Gabrielle Mittaz Hager, Damiano Zemp, Guillaume Roulet, Alice Bridel, Roger Hilfiker

**Affiliations:** 1 Department of Health Professions, Bern University of Applied Sciences, Bern, Bern, 3008, Switzerland; 2 School of Health Sciences, University of Applied Sciences and Arts Western Switzerland, HES-SO Valais-Wallis, Leukerbad, Valais, 3954, Switzerland; 3 Geriatric Service, Ospedale Regionale di Mendrisio (EOC), Mendrisio, Ticino, 6850, Switzerland; 4 Service of Geriatrics and Geriatric Rehabilitation, Lausanne University Hospital (CHUV), Lausanne, Vaud, 1011, Switzerland

**Keywords:** Elderly, Accidental falls, Prevention, Risk assessment

## Abstract

**Background:**

Falls can strongly impact older people’s quality of life, health, and lifestyle. Multifactorial assessment can determine an individual’s risk of falling as the first step for fall prevention intervention. Physiotherapists have an essential role to play in assessing fall risk by older adults living in the community. In the absence of published data on this topic in Switzerland, this study investigated the current practices of physiotherapists to determine whether those are in line with recommendations.

**Methods:**

An anonymous cross-sectional survey was undertaken among physiotherapists practising in Switzerland between the 21st of November and the 31st of December 2020. A priori and exploratory hypotheses were tested. Responses to open-ended questions were grouped into themes for analysis.

**Results:**

A total of 938 questionnaires from all three language regions of Switzerland was analysed. Participants worked in different settings, with a higher representation of private practice self-employees (56%). Standardised fall risk assessments or instruments were used by 580 (62%) participants, while 235 (25%) preferred subjective assessment of fall risk only. Differences in fall risk assessment were observed according to the workplace setting (adjusted OR 1.93, 95% CI 1.37 to 2.7) and education level (trend test, p<0.001). The standardised assessments most frequently employed were the Berg Balance Scale (58%), the Timed-Up-and-Go (57%) and the Tinetti Balance Assessment tool (47%). Risk factors for falls were frequently queried, particularly history of falls (88%), home hazards (84%), and functional ability (81%). Technical resources (40%), knowledge (30%), and time (22%) were common barriers to implement a systematic fall risk assessment.

**Conclusions:**

This study provides an overview of the current practices of physiotherapists in Switzerland in fall risk assessment. There is still room to optimise the standardisation and systematisation of this assessment to implement a best practice strategy and prevent avoidable falls.

## Introduction

The World Health Organization (WHO) estimates that the proportion of people over 60 years old will have doubled by 2050. This age group will account for more than two billion people by 2050, compared to 900 million in 2015 ( World Health Organization, 2018). By general comparison, the Swiss population of older adults has a good general health status ( Merçay, 2017). However, age-related biological changes including but not limited to sarcopenia, reduced abilities in walking, balance and coordination, sight disorders, cognitive decline and comorbidities are risk factors associated with a sharp increase in falls prevalence. For example, in Switzerland, prevalence over the population increased from 21% in 2002 to 26% in 2017 ( Swiss Health Observatory, 2019). Polypharmacy and home hazards may also enhance this risk ( American Geriatrics Society & British Geriatrics Society, 2011; Moreland *et al.*, 2003; Pfortmueller *et al.*, 2014). Worldwide, 28% to 35% of people over 64 years and 32% to 42% of those over 70 suffer of a fall each year. This phenomenon raises with age and level of frailty ( Yoshida, 2007).

Fatal but also non-fatal injuries amongst older adults over 65 years old are mainly attributable to falls ( Houry *et al.*, 2016). Of the 36 million falls in older adults in the United States of America, 20% will have severe consequences such as fractures or head injuries ( Moreland *et al.*, 2020). Those can strongly impact people’s quality of life, health and lifestyle ( Deandrea *et al.*, 2010). Indeed, 21% to 85% of victims of a fall will experience physical, functional, psychological and social changes ( Scheffer *et al.*, 2008). Therefore, a fear of falling can arise, with negative consequences on physical and functional well-being ( Legters, 2002), and a heightened risk of future falls ( Lavedán *et al.*, 2018; Whipple *et al.*, 2018). The falls consequences remain various and can be far-reaching: chronic pain, loss of mobility and autonomy, anxiety, long-term hospitalisations or placements in healthcare centres ( FSO, 2019b; World Health Organization, 2008). This can lead to significant direct financial costs. For example, in Switzerland, hospitalisation for a hip fracture costs CHF 15,000 ( Promotion Santé Suisse, 2016), and a year spent in an institution around CHF 100,000 per person ( AVALEMS, 2019; Promotion Santé Suisse, 2016). In addition, indirect costs, including losses due to premature death, morbidity or disability, unpaid activities, care services, but also intangible costs related to pain and loss of quality of life, are also attributable to falls ( Gannon *et al.*, 2007). Globally, the socio-economic costs (material and immaterial) related to falls amounted to CHF 14.7 billion in 2017 in Switzerland ( BFU *et al.*, 2019).

Guidelines ( American Geriatrics Society & British Geriatrics Society, 2011; Beauchet *et al.*, 2011; Feder *et al.*, 2000; Moreland *et al.*, 2003; National Institute for Health and Care Excellence, 2013), clinical guidance statements ( Avin *et al.*, 2015), and systematic reviews ( Deandrea *et al.*, 2010; Gillespie *et al.*, 2012; Hopewell *et al.*, 2018; Lusardi *et al.*, 2017), have emphasised the effectiveness of multifactorial assessments for determining the risk of falling and for informing the implementation of personalised fall prevention strategies ( Hill, 2009). It is recommended that older adults over 65 years old should regularly (at least once per year) be assessed for fall risk, e.g. by asking about their fall histories, frequencies, contexts and characteristics ( American Geriatrics Society & British Geriatrics Society, 2011).

As movement specialists, physiotherapists constitute a primary point of intervention in preventing falls ( Sherrington & Tiedemann, 2015). They can, for example, inform physicians and home care agencies regarding fall risk factors ( BFU, 2017). Effective fall prevention interventions involve three main steps. First, the screening enables detection of increased fall risk. For example, inquiring regularly about falls history can help to identify individuals at higher risk of future falls. Second, intrinsic and extrinsic factors are investigated through additional assessments such as medication review, mobility level, posture, blood pressure, vision, gait and balance, lower extremity joint function, neurologic and cognitive function, muscular strength, proprioception, reflexes, and/or environmental assessment. Finally, appropriate interventions aim to reduce the rate of falls and the severity of injury. Multidimensional individualised exercise programs are considered a reliable method of preventing falls in older persons ( Hill, 2009; Rubenstein *et al.*, 2001; Montero-Odasso *et al.*, 2022).

However, little is known about the current practices of physiotherapists concerning fall prevention. To our knowledge, there are no published data on the implementation of fall risk assessment guidelines by physiotherapists in Switzerland. To ensure our profession provides guideline-recommended care for older adults, it is necessary to review current clinical practices in screening fall risk in patients over 65 years old.

Therefore, this online survey sought to evaluate to what extent physiotherapists carry out fall risk assessments as the first step towards a fall prevention intervention in the population of community-dwelling older adults in Switzerland. The aim was to determine whether current practices are in line with current recommendations. After identifying barriers and facilitators to fall risk assessment, recommendations on appropriate clinical resources and targeted training will be proposed.

## Methods

### Study design

A cross-sectional survey of physiotherapists currently practising in Switzerland was undertaken between the 21st of November and the 31st of December 2020.

### Participants

Registered physiotherapists working in Switzerland and providing care to patients over the age of 65 in their daily practice were eligible to participate. Two mandatory screening questions were used to confirm eligibility: *i) Are you currently (or in the last 12 months) working as a physiotherapist in Switzerland? ii) Are you managing patients over 65, regardless of their initial pathology?*

*Recruitment strategy*

In the absence of a federal register comprising all currently practising physiotherapists in Switzerland, a comprehensive recruitment strategy was developed by the research team. It was designed to optimise the results generalisability and sample representativeness. Therefore, a range of organisations was asked to assist with study recruitment by sending the questionnaire to their members, which maintained confidentiality. First, all Swiss cantonal physiotherapy associations were invited to participate. Sending confirmations were received from Bern, Valais, Basel, Neuchâtel, and Aargau organisations. Additionally, the Swiss Association of Independent Physiotherapists (ASPI-SVFP) and the Swiss Association of Sports Physiotherapy (Sportfisio) also agreed to send the study questionnaire to their members. To broaden the sampling frame, Master’s students in physiotherapy at the Bern University of Applied Sciences (BFH) and the Zürich University of Applied Sciences (ZHAW), as well as ALUMNI physiotherapists from the HES-SO Valais-Wallis, were invited to participate. Moreover, lists of physiotherapy practices (n=2000) for each of the 26 Swiss cantons were created manually with online research on local.ch. Finally, the questionnaire was sent to all physiotherapists working on the Swiss CHEF Trial project. This ongoing national randomized controlled trial compares three home-based exercises programs aiming at preventing falls in older people of Switzerland ( Mittaz Hager *et al.*, 2019).

*Non-monetary incentives*

To maximise responses, all the participants who took part in the survey had the opportunity to participate in a prize draw ( Andrews *et al.*, 2003; Edwards *et al.*, 2009; Tuten *et al.*, 2000). Three prizes were offered, consisting of an overnight hotel stay. To ensure the anonymity of the survey responses, a separate webpage was used to collect and store the email addresses. There was no link between survey responses and email addresses stored for the prize draw.

### Survey development

A draft questionnaire was developed using an iterative process with the research team, which included six physiotherapists with several years of experience in fall prevention and geriatrics. Permission to use clinical practice questionnaires developed for similar studies was obtained from two authors ( Ackerman *et al.*, 2019; Gaboreau *et al.*, 2016). Ackerman *et al.* targeted fall prevention by older people with osteoarthritis and Gaboreau *et al.* focussed their study on general practitioners’ (GPs) routines linked to this topic. Their questionnaires were partially adapted to align with the research question and Swiss healthcare context. To maximise study quality and research rigour, the Checklist for Reporting Results of Internet E-Surveys (CHERRIES, see extended data “CHERRIES Checklist” ( Duc *et al.*, 2022)) ( Eysenbach, 2004), results of different guidelines, systematic reviews and studies ( Andrews *et al.*, 2003; Artino *et al.*, 2018; Edwards *et al.*, 2009; Kelley *et al.*, 2003; Lumsden & Morgan, 2005) as well as websites ( Creative Research Systems, 2016; Deckers, 2017; Parrott, 2020) were followed. See extended data “Designing Tools” for brief descriptions of each of the tools mentioned above ( Duc *et al.*, 2022).

The semi-structured online questionnaire consisted of 52 short questions with several branching logics (adaptive questioning). This process allowed some questions to be displayed conditionally based on the answers to prior items, intentionally reducing the responder burden. The survey included a mix of open-ended questions and multiple response options covering first participants characteristics. As no identifying information was collected, the anonymity of the participants was preserved. Workplace, assessed only with the first two postal code numbers, was used to observe the presence of falls prevention programmes by geographical area. Perceived ability to assess and manage older patients was evaluated on two purposed-designed Likert scales ranging from 0 “none” to 100 “excellent”. The responsibility of nine health care professions in fall risk assessment was also questioned on Likert scales ranging from 0 “not at all concerned” to 10 “very concerned”. The way physiotherapists screen for fall risk included questions about the situations leading to testing the patient, the tests used, and the interventions undertaken. Risk factors and the way they are measured were also evaluated. One question specifically targeted the reasons why some therapists never assess fall risk to understand barriers to fall risk assessment. How physiotherapists quantified the risk of falling, reassessed patients and the elements needed to facilitate a more systematic risk assessment were asked at the end of the questionnaire. Three hypothetical patient cases (vignettes) from the musculoskeletal, respiratory, and neurological fields were also presented to respondents and their management related to fall risk assessment evaluated.

The questionnaire was first developed in French and English. It was translated entirely into three of the four country's main languages, French, German and Italian, to send it throughout Switzerland. As Romansh speakers represent only 0.5% of the total population ( FSO, 2019a) and are primarily fluent in German or Italian ( FSO, 2020), it was estimated that they could use one of those versions. The forwards translation was completed by the authors with the help of two specialised companies. The survey is also supplied in English in the extended data (see “Survey”) ( Duc *et al.*, 2022).

For ease of reading, no more than three questions were presented per page. At any time, respondents could review and change their answers using the “previous” button functionality. In most cases, the “other” option allowed participants to indicate a missing response option if necessary. Physiotherapists were free to terminate their participation definitively or momentarily at any time. They could proceed to the next page or skip questions. Except for the two screening questions, only one question was mandatory: “ *Do you usually use fall risk screening tools when assessing your patients over 65 years of age?”.* By submitting their email address, the server automatically generated a link allowing participants to receive a partially completed questionnaire as is so that they could complete it later. See extended data “Survey Development” for a short description of the development stages of this questionnaire ( Duc *et al.*, 2022).

### Survey validation

*Ace Validity Testing and Preliminary Pilot Testing*

We searched the literature and contacted experts to define and conceptualise relevant topics for the survey item generation. The different steps of survey validation were conducted with the help of 21 physiotherapists who were experienced in the treatment of older adults and measurement properties.

Questionnaire feasibility was assessed, as well as content and face validity and comprehensibility. Dillman developed a four-step methodological pre-testing process adapted and applied for this study to assess validity according to Artino *et al.* recommendations ( Artino *et al.*, 2018; Dillman, 2000). Refinements were made at each stage as required. In extended data “Survey Pre-testing and Validation” summary tables of Dillman’s pre-testing steps and their application in this study are presented. Questions used for survey comprehensibility assessment are provided in extended data “Comprehensibility Assessment” ( Duc *et al.*, 2022). The answers collected during the pre-testing and validation process were deleted before the questionnaire went online.

*Assessment of reliability*

Reliability and validity are essential scores to consider when elaborating a questionnaire ( Artino *et al.*, 2018). Validity assessment was undertaken following the pre-test process of Dillman ( Dillman, 2000), and modifications were undertaken if necessary. To meet the 31st of December 2020 deadline for completion of data collection, this step was first not evaluated. However, test-retest reliability could be assessed between November 2021 and January 2022 for the publication of this work. The assessment of reliability was the final step in the survey validation. The questionnaire was sent to seventeen French and German speaking physiotherapists who took part twice to assess the test-retest reliability. Intraclass Correlation Coefficient (ICC) was measured for non-dichotomous data using ICC _2,1_ and Cohen's unweighted kappa was calculated to estimate test-retest reliability of dichotomous items ( Streiner *et al.*, 2015). The lower limit values indicating moderate reliability were 0.41 for the kappa ( Bland, 1991) and 0.7 for the ICC ( Streiner *et al.*, 2015).

### A priori hypotheses

A set of 18 a priori hypotheses was established before the start of the data collection. These are presented in extended data “A-priori Hypotheses” ( Duc *et al.*, 2022). Hypotheses that emerged after the end of data collection were specified as explorative.

### Project website

Consistent with the study’s main objectives, a website was specially designed. The survey was available in the three translation languages (French, German and Italian) and contained the key elements of the study organised by subthemes. An anonymous chat provided by Crisp allowed participants to ask questions and obtain almost instantaneous help. No IP tracking or cookies have either been used to ensure the anonymity of individuals visiting this website. The final report of this work will be published on this website for interested participants to access.

### Data collection

On the 21st of November 2020, physiotherapists received an email with a link to the online survey hosted on the REDCap server of the HES-SO. Physiotherapists could contact the research team at any time for further information, either by email or anonymously via a chat on the project website. To avoid transcription errors, responses were immediately sent to the project’s password-protected REDCap database ( Andrews *et al.*, 2003). One reminder was sent to all participants three weeks after the initial mailing (i.e. 08.12.2020) to increase response rates ( Andrews *et al.*, 2003; Edwards *et al.*, 2009; Lau, 2017). Data collection was completed by the 31st of December 2020. All details regarding survey distribution and follow-up are described in extended data “Survey Send Out” ( Duc *et al.*, 2022).

The cover letter gave an overview of the study's main objectives and efforts to preserve participant confidentiality and anonymity (see extended data “Cover Letter” ( Duc *et al.*, 2022)). It was specified that by submitting the two eligibility assessment questions, informed consent was implied. Eligible participants were led to the survey, and those considered ineligible were informed that they could not take part. Participation could involve any electronic device, and the estimated duration did not exceed 25 minutes. At no time were an IP tracking or cookies used. No other techniques to analyse the log file for the identification of multiple entries were used. The link to the online survey was posted on the project website but no other advertisement was done.

### Ethical approval

An approval of Swiss Ethics was obtained via the “Clarification of responsibility” form on the portal of the Business Administration System for Ethics Committees (BASEC) in May 2020. As the survey was anonymous and no health-related data were asked, this study did not fall under the Swiss Human Research Act (CER-VD Req-2020-00515).

### Data analysis

Data were analysed with R, version 4.0.5 ( R: The R Project for Statistical Computing, 2020), RStudio, version 1.4.1106 ( RStudio|Open Source & Professional Software for Data Science Teams, 2020) and Stata (Version 17, StataCorp, College Station, Texas) ( Stata: Software for Statistics and Data Science, 2020).

*Handling of variable’s grouping*

When evaluating how physiotherapists assess the fall risk of their patients, of the 938 participants, only 8 (0,1%) reported that they never assess the fall risk. Therefore, it was decided not to use this item to test the hypotheses. Instead, a binary variable “use standardised assessment” was created from the four response options: *i) By a subjective evaluation (observation of the patient, discussion…), ii) By a standardised evaluation (scale, functional tests, questionnaires, timed tests…) iii) By instrumental assessment (inertial sensors, force platforms, EMG…) iv) I do not perform an assessment.* We dichotomised them as follows: “use standardised assessment”: yes = ii) & iii), no = i) & iv).

We categorised the year of completion of the highest education level as follows: i) “Before 1990”: < 1990, ii) “1990 to 1999”: > 1990 & < 2000, iii) “2000 to 2009”: > 2000 & < 2010, iii) “2010 or later”: > 2010.

A binary variable “Clinic or Private Practice” was created from the different work settings: *i) Hospital, ii) Rehabilitation clinic, iii) Private practice as employee, iv) Private practice as self-employed person, v) Retirement home, vi) Home-based physiotherapist, vi) Other(s).* We dichotomised them as follows: “Institution” = i) & ii) & v), “Private practice” = iii) & iv) & v) & vi).

Vignettes of patients with musculoskeletal, neurological, or respiratory problems were presented: *i) A 65-year-old man consults for rheumatological problems (e.g., Osteoarthritis). His physiotherapy referral mentions "anti-inflammatory analgesia, improvement of joint and muscle function". What do you do?*

ii) A 72-year-old woman consults because of neurological problems (e.g., Parkinson's disease). Her physiotherapy referral mentions "improvement of joint and muscle function, proprioception/coordination". What do you do?

iii) A 68-year-old man consults because of respiratory disease (e.g., Chronic Obstructive Pulmonary Disease). His physiotherapy referral mentions "improvement of cardiopulmonary function". What do you do?

The response options for the three vignettes were: *i) I complete my assessment by using a specific fall risk assessment tool, ii) I do not complete my assessment by using a specific fall risk assessment tool, but I assess the main factors such as muscle strength, static and dynamic balance…, iii) As the patient does not consult for a fall risk problem, I do not assess either his risk or his risk factors for falling, iv) I do not treat this type of pathology in my daily practice.* For some analyses, we dichotomised them as follows: “does fall risk assessment”= i) & ii), “does no fall risk assessment” = iii). The last answer option (iv) was excluded from this analysis.

*Statistics*

The distributions of the continuous variables were analysed visually, and median, interquartile range, minimal and maximal values were reported. Categorical variables were summarised with absolute and relative frequencies. 95% confidence intervals were reported to document the statistical precision of the estimates. The significance level was set at p<0.05 for all tests.

Differences between participants with more than 10% missing values and those with fewer missing values were compared with non-parametric tests for continuous variables and with Chi-squared tests for frequency tables. Cohen’s d effect size was calculated for continuous variables. An effect size of 0.2 was considered a slight difference, 0.5 a moderate difference and 0.8 a significant difference. Cramer’s V effect sizes were calculated for categorical variables, where 0.1 was considered small, 0.3 moderate and 0.5 large.

The hypotheses were tested using multivariable logistic regression for binary dependent variables and Chi-squared tests for categorical dependent variables. The logistical and linear regressions were adjusted for a set of potential confounders. Variables for the multivariable models were selected using Directed Acyclic Graphs (DAGs) according to theoretical considerations ( VanderWeele, 2019). Nonparametric Cochran-Armitage statistics was used to test trend (i.e., hypothesis 2, increasing proportion of physiotherapists assessing with increasing education level). Contrast after an ANOVA was used to test whether the responsibility for screening was higher for physiotherapists as for other professions (hypothesis 5).

An additional exploratory analysis was performed. i.e., it has been clearly stated that no a priori hypothesis was formulated and that this result needed to be interpreted with caution.

The textual content of the semi-open questions (n=13) was analysed through a content analysis using an inductive approach ( Elo & Kyngäs, 2008; Graneheim & Lundman, 2004). This analysis approach was the most feasible due to the extensive data and the human resources available. Each free text response (meaning unit) was closely reviewed and summarised into main concepts (condensed meaning units). Those were then grouped into emerging themes making clinical or theoretical sense. To illustrate them, some participants' responses are provided verbatim in their original language.

Finally, because there was some evidence of differences regarding fall risk activities and admission patterns to nursing home according to Swiss cantons ( Merçay, 2017), it was decided to present the descriptive results stratified per language.

### Response ratio

According to the Swiss Federal Statistical Office (FSO), it was impossible to know the official number of physiotherapists currently practising in Switzerland. Because third parties sent invitation emails, we do not know the number of emails delivered. Therefore, the response ratio could not be calculated.

### Sample size considerations

The members of the national physiotherapy association, Physioswiss (n=10’652 in December 2019), and the Swiss Association of Independent Physiotherapists, ASPI-SVFP (n=500 in December 2019) were added to estimate the Swiss population of working physiotherapists, acknowledging that not all physiotherapists are members of one of these associations or that a potential overlap might occur. With an estimated precision of 5% (i.e. 95% confidence interval span of 10%) and an estimated population of 11'155 individuals, complete answers of 371 therapists were required ( The Survey System). This calculation required the assumption of random sampling, i.e., that our sample is representative of all physiotherapists working in Switzerland. However, we were not able to test this assumption.

### Atypical timestamps

The timestamps associated with questionnaire completion was analysed for each complete questionnaire (submitted or the last question answered) and partially complete questionnaires (not submitted or the last question not answered), all having a completeness ratio ≥ 90%. Data from participants who responded too quickly (i.e., < 5 minutes) were excluded from the analyses. Indeed, as the average time to take part in the questionnaire was estimated at 25 minutes, 5 minutes was not considered a serious attempt ( Huang *et al.*, 2012).

### Completion ratio and missing values

The survey completion ratio was calculated by dividing the number of respondents who submitted the survey or completed the last question per the number of those who completed the informed consent.

Participant could skip questions if they did not want to respond. Only the inclusion criteria and the screening frequency were set as mandatory. The proportion of missing values per question, and the proportion of missing responses per participant, were calculated considering the branching logics (adaptive questioning). For sensitivity analyses, we analysed the participants with more than 10% missing responses and with less than five minutes completion time separately and compared the results to the other participants with statistical tests and effect sizes (Cohen’s d for continuous variables and Cramer’s V for categorical variables).

### Participation ratio

It was intended that study participants should remain anonymous. Therefore, no IP tracking or cookies were used. Hence, it was impossible to know who clicked on the survey invitation link. In addition, due to data protection reasons and the anonymous survey, it was impossible to identify a unique visitor or to test whether a person responded twice. Consequently, the participation ratio (unique participants responding to the survey divided by those clicking on the invitation link) cannot be calculated.

## Results

### Reliability

The median ICC (for the ordinal and continuous variables) was 0.86, with only one item below 0.7. The median Kappa value for the dichotomous items was 0.65, with 15% of items with Kappa values below 0.41. The items with lower than moderate Kappa values (i.e. <0.41) were items with many multiple choice response options (e.g. the type of falls screening tool used, the advice given regarding falls risk the type of balance assessments used). Furthermore, the responses for the question about which professionals are responsible for the screening of falls risk, the results for the nurses and occupational therapist had too low Kappa values.

### Survey responses

**Figure 1.**
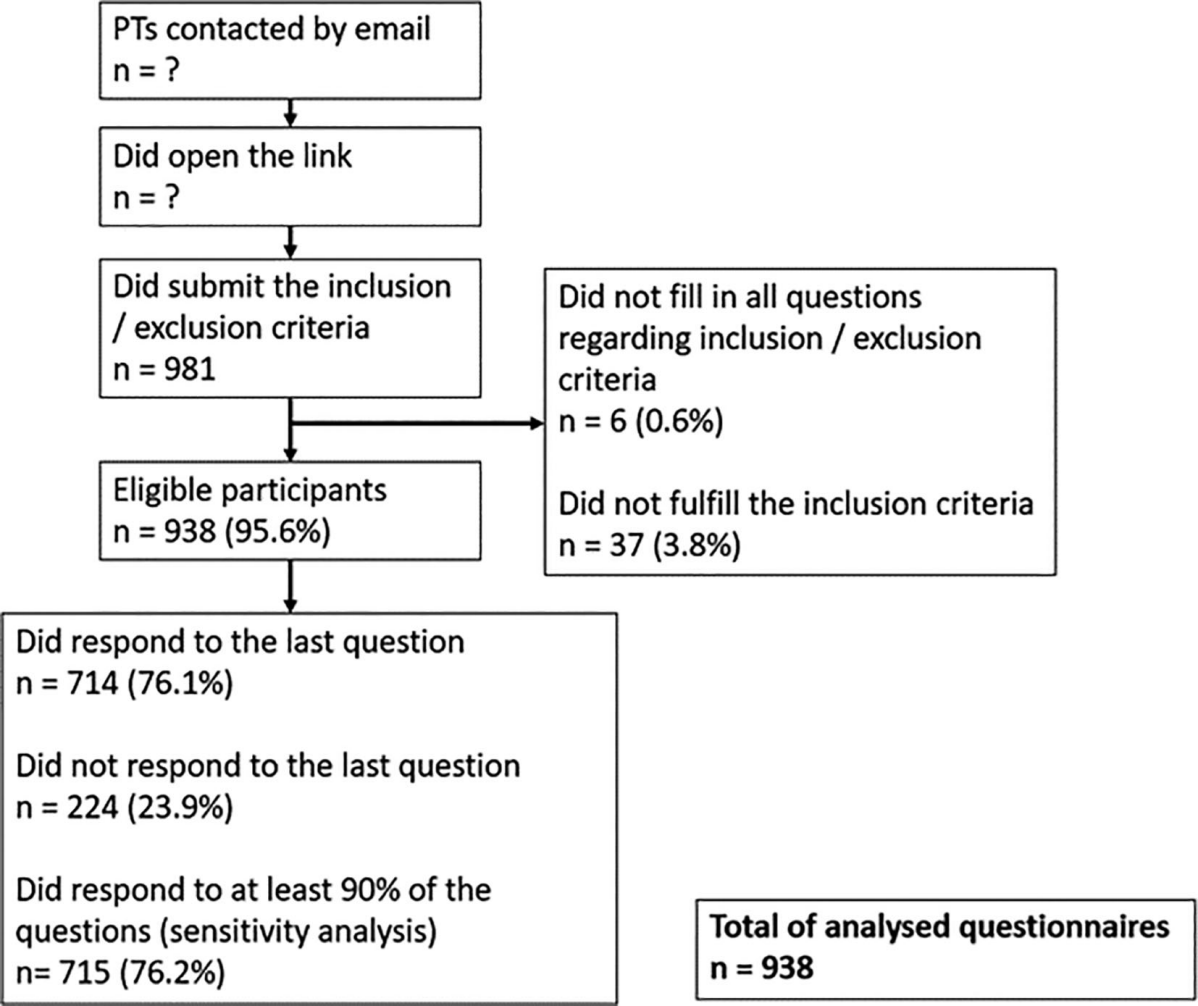
Survey flow chart. PTs = physiotherapists, n = number. ? = Number unknown because i) associations did not reveal the number of emails sent, ii) overlap of emails sent (physiotherapists may be in different mailing lists). For the percentages: 100% corresponds to the 938 eligible physiotherapists, except for the percentages in the box of the eligible participants, where 100% corresponds to the 981 participants who submitted the responses on the inclusion and exclusion criteria.

A total of 981 individuals completed the two screening questions to assess their eligibility. Among them, 938 (95.6%) were eligible to participate. 715 (76.2%) physiotherapists (PTs) responded to at least 90% of the questions, 714 (76.1%) to the last question and 224 (23.9%) excited the survey prematurely, only providing partial data. All questionnaire data, even partially completed, were imported, analysed, and the attrition ratio (number of missing values per item) taken into consideration. The participants flow chart is presented in Figure 1.

### Atypical timestamps

The median participation time was 17 minutes (IQR 11 to 27), and no participant data were excluded because of atypical time required to complete the questionnaire.

### Completion ratio and missing values

The completion ratio for this study was 76.1%. The percentage of missing responses per items ranged from 0 to 24% (see extended data “Missing Values” ( Duc *et al.*, 2022)).

The sensitivity analyses with the comparison of those with more than 10% missing responses to those with fewer missing responses showed several differences between both groups. Participants with more missing responses were three years older (Cohen’s d of 0.24, p<0.05) and less frequently had an education with a bachelor’s degree or higher (Cramer’s V effect size 0.13, p<0.05). Moreover, they also had 10% more participants who received their diploma before 1990 (p<0.05), 12% more who worked in private practice as employees (Cramer’s V effect size 0.13, p<0.05), and 7% less who worked in hospitals (Cramer’s V effect size 0.09, p<0.05). Furthermore, those with more missing values reported less confidence in the assessment of the fall risk (Cohen’s d 0.25, p<0.05) and less confidence in the management of people with an increased fall risk (Cohen’s d 0.19, p<0.05), compared to those with less missing values. Because of these systematic differences, it was decided not to exclude those with more than 10% missing as it would induce a sampling bias.

### Participants’ socio-demographic characteristics

The characteristics of the respondents are shown in Table 1.

**Table 1.** Demographic and professional characteristics of the participants.

	French (N=342)	German (N=531)	Italian (N=65)	Total (N=938)
**Age (years)**				
- Count	336	509	62	907
- Median (IQR)	40.00 (30.00, 55.00)	47.00 (36.00, 56.00)	41.50 (35.00, 52.75)	44.00 (33.00, 56.00)
**Gender**				
- Men	112 (33.1%)	137 (26.6%)	29 (46.0%)	278 (30.3%)
- Women	223 (66.0%)	379 (73.4%)	33 (52.4%)	635 (69.2%)
- I do not wish to specify	3 (0.9%)	0 (0.0%)	1 (1.6%)	4 (0.4%)
**Highest education level**				
- Specialised School (without bachelor’s degree)	98 (30.4%)	208 (40.7%)	15 (24.6%)	321 (35.9%)
- Bachelor’s degree (BSc)	125 (38.8%)	166 (32.5%)	19 (31.1%)	310 (34.7%)
- Certificate of Advanced Studies (CAS)	43 (13.4%)	35 (6.8%)	15 (24.6%)	93 (10.4%)
- Diploma of Advanced Studies (DAS)	9 (2.8%)	3 (0.6%)	1 (1.6%)	13 (1.5%)
- Master of Advanced Studies (MAS, 60 ECTS)	13 (4.0%)	24 (4.7%)	3 (4.9%)	40 (4.5%)
- Master of Sciences in Physiotherapy (MSc, 90 ECTS)	33 (10.2%)	71 (13.9%)	6 (9.8%)	110 (12.3%)
- PhD	1 (0.3%)	1 (0.2%)	0 (0.0%)	2 (0.2%)
- Other	0 (0.0%)	3 (0.6%)	2 (3.3%)	5 (0.6%)
**Graduation year**				
- Before 1990	58 (18.4%)	107 (21.3%)	7 (12.5%)	172 (19.7%)
- 1990 to 1999	38 (12.1%)	76 (15.1%)	9 (16.1%)	123 (14.1%)
- 2000 to 2009	64 (20.3%)	106 (21.1%)	13 (23.2%)	183 (20.9%)
- 2010 or later	155 (49.2%)	214 (42.5%)	27 (48.2%)	396 (45.3%)
**Practice setting**				
- Hospital	36 (10.5%)	82 (15.4%)	2 (3.1%)	120 (12.8%)
- Rehabilitation clinic	28 (8.2%)	23 (4.3%)	1 (1.5%)	52 (5.5%)
- Private practice as an employee	74 (21.6%)	110 (20.7%)	16 (24.6%)	200 (21.3%)
- Private practice as a self-employed person	180 (52.6%)	302 (56.9%)	43 (66.2%)	525 (56.0%)
- Retirement home	27 (7.9%)	78 (14.7%)	8 (12.3%)	113 (12.0%)
- Home-based physiotherapist	98 (28.7%)	99 (18.6%)	28 (43.1%)	225 (24.0%)
- Other	4 (1.2%)	6 (1.1%)	1 (1.5%)	11 (1.2%)
**Work setting**				
- Work alone	223 (68.6%)	283 (55.9%)	38 (61.3%)	544 (60.9%)
- Work in team	102 (31.4%)	223 (44.1%)	24 (38.7%)	349 (39.1%)
- Only with other physiotherapists	30 (30.0%)	106 (48.0%)	11 (45.8%)	147 (42.6%)
- With other health professionals	70 (70.0%)	115 (52.0%)	13 (54.2%)	198 (57.4%)
**Part-time working**	190 (59.4%)	319 (63.9%)	27 (45.0%)	536 (61.0%)
**Work percentages**				
- Count	187	286	27	500
- Median (IQR)	70.00 (50.00, 80.00)	60.00 (50.00, 73.75)	60.00 (50.00, 80.00)	60.00 (50.00, 80.00)
**Specific training in geriatrics**	69 (21.2%)	186 (36.7%)	19 (30.6%)	274 (30.6%)
**Older adults treated per week**				
- Count	311	478	61	850
- Median (IQR)	14.40 (8.00, 20.00)	13.71 (8.00, 20.00)	14.00 (9.60, 20.00)	14.00 (8.00, 20.00)
**Physiotherapy referrals to reduce the risk of falls received within 12 months**				
- 0 to 5	124 (50.0%)	196 (54.1%)	28 (59.6%)	348 (53.0%)
- 6 to 10	51 (20.6%)	76 (21.0%)	10 (21.3%)	137 (20.9%)
- 11 to 15	18 (7.3%)	26 (7.2%)	2 (4.3%)	46 (7.0%)
- 16 and more	55 (22.2%)	64 (17.7%)	7 (14.9%)	126 (19.2%)
**Confidence level for assessing the risk of falls**				
- Count	306	469	55	830
- Median (IQR)	74.00 (61.00, 80.00)	75.00 (60.00, 85.00)	76.00 (69.50, 83.50)	75.00 (60.00, 83.00)
**Confidence level in managing older patients at risk of falling**				
- Count	304	460	56	820
- Median (IQR)	80.00 (70.00, 90.00)	75.00 (60.00, 85.00)	80.00 (69.00, 90.00)	79.00 (65.00, 87.00)

IQR = interquartile range; PhD = Doctor of Philosophy.

**Figure 2.**
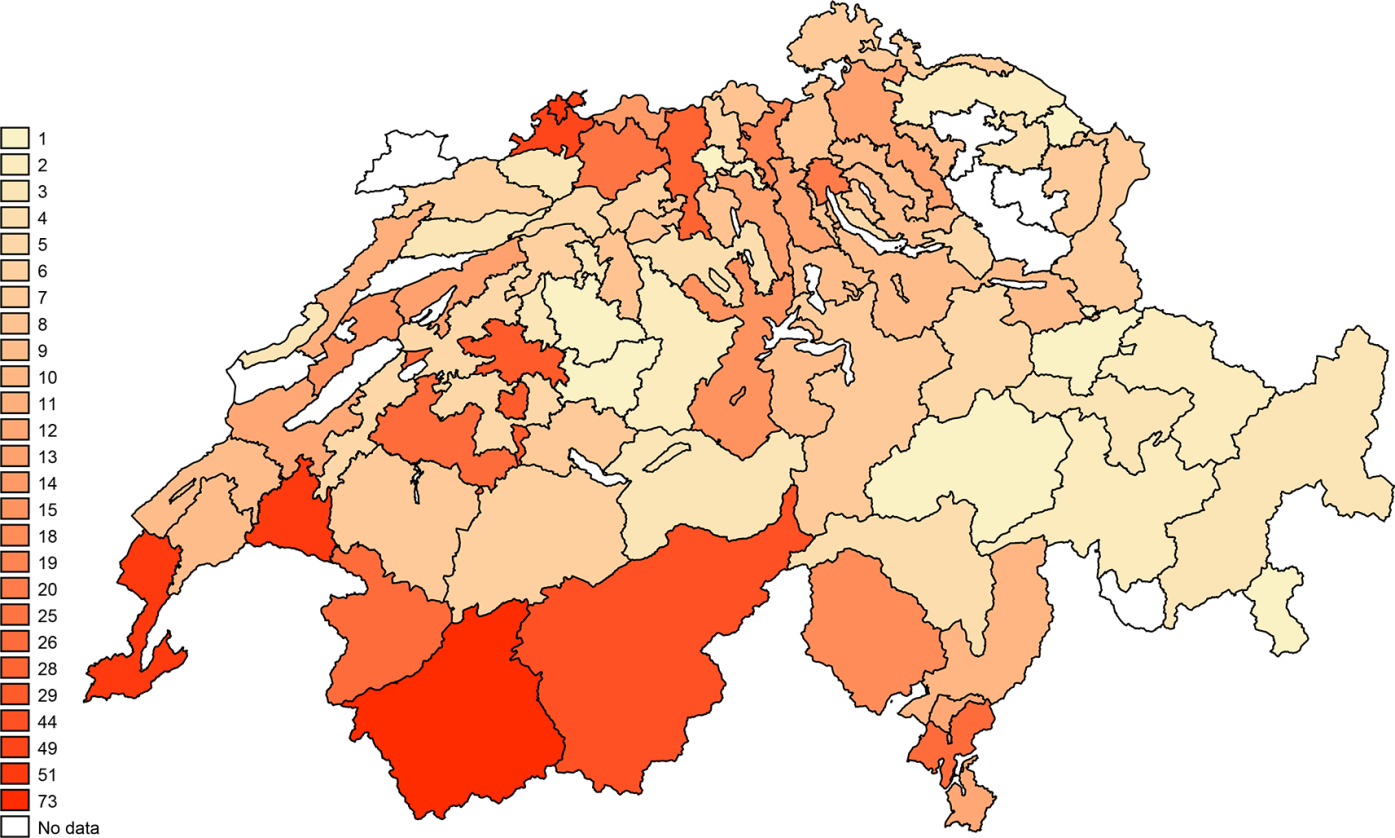
Number of participants per two-digits zip-code zones. The numbers correspond to the number of participants in each two-digits zip-code zone.

Most PTs were women (n=635, 69.2%), and the median age was 44 years (IQR 33 to 56). Participants came from all three language regions of Switzerland (see Figure 2). Most of the respondents were either graduates of a specialised school (n=321, 35.9%) or had a bachelor’s degree (n=310, 34.7%). Moreover, the most frequent formal training was a Master of Sciences in Physiotherapy (n=110, 12.3%). Several participants also had specific training in geriatrics (n=274, 30.6%). Certificates attesting to the highest education level were obtained after 2010 for 396 PTs (45.3%).

**Figure 3.**
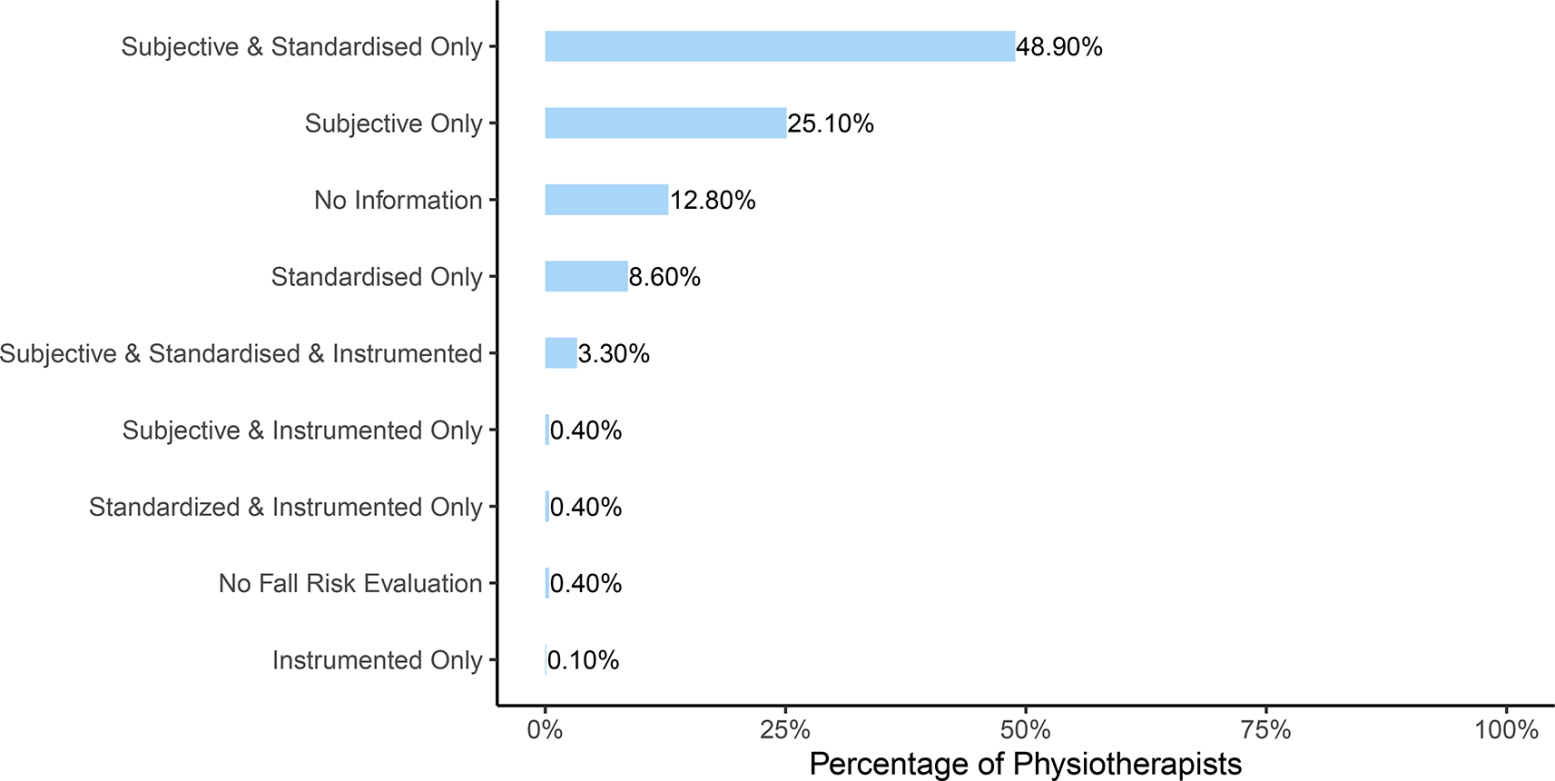
Distribution of mutually exclusives categories of assessments used by the physiotherapists. Percentage of physiotherapists using a specific category of assessments.

The participants worked in different settings with a high representation of those in private practice as self-employee (n=525, 56%). Of the 349 (39.1%) respondents working in a team, 198 (57.4%) were in a multidisciplinary team, including other health professionals. Most participants (n=536, 61%) worked part-time with a median of 60% (IQR 50 to 80).

The median number of patients over 65 years of age treated per week by the respondents was 14 (IQR 8 to 20). Within 12 months, about 53 % (n=348) of PTs received less than 5 referrals to reduce the fall risk, 21% (n=137) got between 6 and 10 referrals and 19.2% (n=126) more than 16. Finally, on a Likert scale ranging from 0 “none” and 100 “excellent”, the participant’s median confidence level in assessing the risk of falls in older adults was 75 (IQR 60 to 83) and 79 (IQR 65 to 87) in managing a patient at risk.

### A priori hypotheses analysis

Of the 18 a priori hypotheses formulated before the questionnaire was sent to the participants, two were merged during the analyses because of their similarity (17 and 18). The set of hypotheses as initially drafted is presented in extended data “A-priori Hypotheses” ( Duc *et al.*, 2022). The summarised results of each hypothesis are provided in Table 3. Details of the descriptive statistics of the variables related to these hypotheses can be found in Tables 4 to 11.

**Table 2.** Fall risk assessment frequencies according work setting.

Use of standardised instrument	Private practice n (%)	Institution n (%)	Missing
Never	188 (30%)	33 (12.2%)	0 (0)
Sometimes (1% to 49% of patients)	229 (36.5%)	80 (29.6%)	1 (2.4%)
Often (50% to 94% of patients)	129 (20.6%)	96 (35.6%)	0 (0%)
Always (95% to 100% of patients)	25 (4%)	38 (14.1%)	0 (0%)
Missing responses	56 (8.9%)	23 (8.5%)	40 (97.6%)
**Total**	627 (100%)	270 (100%)	41 (100%)

**Table 3.** Statistical results for the a-priori defined hypotheses.

H	Hypotheses	Statistical tests	95% CI	Missing values	Hypothesis
1a	PTs in institution (hospital, rehabilitation clinic or retirement home) use standardised assessment more than independent PTs (private practice, at home).	OR crude 2.02 adjusted 1.93	1.47; 2.78 1.37; 2.7	41 63	Confirmed
1b	PTs in institution assess fall risk more frequently than independent PTs.	X ^2^ 70.8662, df 3, p <0.001 OR crude 3.15 adjusted 2.85	2.09; 4.75 1.86; 4.38	41	Confirmed
1c	PTs in institution assess fall risk more often in musculoskeletal patients than independent PTs.	OR crude 2.06 adjusted 2.02	1.05; 4.04 1.01; 4.04	239 254	Confirmed
1d	PTs in institution assess fall risk more often in neurological patients than independent PTs.	RR ‡ crude 1.01 adjusted 1.02	0.86; 1.19 0,86; 1.20	273 286	Not confirmed
1e	PTs in institution assess fall risk more often in respiratory patients than independent PTs.	OR crude 1.61 adjusted 1.51	1.07; 2.44 0,98; 2.33	358 371	Not confirmed
2	PTs with higher education level use more often a standardised assessment than others.	Test for trend p<0.001		44	Confirmed
3	Part-time PTs assess fall risk less than full-time PTs.	OR crude 1.12 adjusted 1.18	0.85; 1.49 0.48; 1.65	59 80	Not confirmed
4	PTs working alone use a standardised assessment less than those in a team.	OR crude 0.44 adjusted 0.52	0.33; 0.59 0.37; 0.72	45 66	Confirmed
5	PTs assessing fall risk think that it is primarily the responsibility of their profession.	Contrast/ANOVA p <0.001 ( Figure 4)		194	Confirmed
6	The main reasons why PTs do not assess fall risk are the lack of time, forgetting and not having a role to play.	Proportions p>0.05 that lack of time is the main reason. ( Figure 6 and Table 5)		0	Not confirmed
7	Therapists are mostly pressured to conduce a fall risk assessment if clear signals of a risk of falling are mentioned such as the the history of falls and receiving a specific referral.	Proportions p<0.001 that fall history is the most frequently mentioned reason. p>0.05 that specific referral is the most frequently mentioned reason. ( Figure 5 and Table 6)		0	Partially confirmed
8	PTs who assess fall risk more often also advise more patients on risk factors.	Proportions RR “often” vs “never”: 1.22 (95% CI 1.12 to 1.33) ( Table 7)		119	Confirmed
9	PTs who regularly perform fall risk assessment use a specific tool more often than others.	Proportions RR “often” vs “never”: 1.42 (95% CI 1.28 to 1.59) ( Table 8)		0	Partially confirmed
10	PTs focus mainly on fall history, muscle strength and balance when assessing the risk factors of falls.	Proportions 89% (95% CI 0.86 to 0.91) assess fall history, muscle strength or balance but they also assess other aspects as frequently. ( Table 9)		196 to 207	Not confirmed
11	PTs who regularly perform fall risk assessment conduct re-evaluations of all their patients over 65 years old more often than others.	X ^2^ 8.9432, df 2, p = 0.0114		73	Confirmed
12	PTs performing frequently fall risk assessment conduct re-evaluations of all their patients assessed as being at risk of falling more regularly than others.	Linear regression crude Always vs. Sometimes: 7.56 (3.56; 11.55) adjusted Always vs. Sometimes: 4.48 (0.3; 8.65)		205 223	Confirmed
13	PTs detecting a risk of falling in their patients will mainly inform them of the situation directly and give them some exercises to do at home.	Proportions p<0.001 (count regression) ( Table 10)		119	Confirmed
14	PTs using a standardised tool mostly quantify their patients' risk of falling because this procedure is intrinsic to these assessment tools.	OR crude 4.68 adjusted 4.24	3.11; 7.04 2.70; 6.65	210 241	Confirmed
15	Most PTs quantifying their patients' risk of falls do so by dichotomization (risk/no risk).	Proportions p>0.05 ( Table 4)		210	Not confirmed
16	More of 50% of PTs state that systematic fall risk assessment (= of every new patient over 65 years of age) should be performed.	Proportions p>0.05 ( Table 4)		222	Not confirmed
17	60% PTs state that they would need a quick and easy tool to facilitate a systematic assessment.	Proportions p>0.05 ( Table 11)		557	Not confirmed

OR = odds ratio; CI = confidence interval.

‡ Due to a cell with 0, the calculation of odds ratio was not feasible, therefore the risk ratio is reported here.

**Table 4.** Results of the hypothesis testing regarding screening approaches.

	French (N=342)	German (N=531)	Italian (N=65)	Total (N=938)
**How PTs assess patients over 65**				
- I do not perform a fall risk assessment	1 (0.3%)	7 (1.3%)	0 (0.0%)	8 (0.9%)
- By subjective assessment	275 (80.4%)	409 (77.0%)	45 (69.2%)	729 (77.7%)
- By a standardised assessment	218 (63.7%)	318 (59.9%)	39 (60.0%)	575 (61.3%)
- By instrumental assessment	12 (3.5%)	26 (4.9%)	2 (3.1%)	40 (4.3%)
**Standardised fall risk assessment**				
- Yes	220 (64.3%)	321 (60.5%)	39 (60.0%)	580 (61.8%)
- No	122 (35.7%)	210 (39.5%)	26 (40.0%)	358 (38.2%)
- Missing	0 (0.0%)	0 (0.0%)	0 (0.0%)	0 (0.0%)
**How PTs assess musculoskeletal patients**	**N=253**	**N=416**	**N=50**	**N=719**
- I complete my assessment using a specific fall risk assessment tool	41 (16.2%)	38 (9.1%)	0 (0.0%)	79 (11.0%)
- I do not complete my assessment with a specific fall risk assessment tool but I assess the main risk factors	195 (77.1%)	324 (77.9%)	42 (84.0%)	561 (78.0%)
- As the patient does not consult for a fall risk problem, I do not assess his risk, or his fall risk factors	11 (4.3%)	41 (9.9%)	8 (16.0%)	60 (8.3%)
- I do not manage this type of pathology in my daily practice	6 (2.4%)	13 (3.1%)	0 (0.0%)	19 (2.6%)
**How PTs assess neurological patients**	**N=252**	**N=413**	**N=49**	**N=714**
- I complete my assessment using a specific fall risk assessment tool	121 (48.0%)	184 (44.6%)	17 (34.7%)	322 (45.1%)
- I do not complete my assessment with a specific fall risk assessment tool but I assess the main risk factors	114 (45.2%)	194 (47.0%)	30 (61.2%)	338 (47.3%)
- As the patient does not consult for a fall risk problem, I do not assess his risk, or his fall risk factors	0 (0.0%)	6 (1.5%)	0 (0.0%)	6 (0.8%)
- I do not manage this type of pathology in my daily practice	17 (6.7%)	29 (7.0%)	2 (4.1%)	48 (6.7%)
**How PTs assess respiratory patients**	**N=251**	**N=412**	**N=50**	**N=713**
- I complete my assessment using a specific fall risk assessment tool	26 (10.4%)	29 (7.0%)	3 (6.0%)	58 (8.1%)
- I do not complete my assessment with a specific fall risk assessment tool but I assess the main risk factors	126 (50.2%)	214 (51.9%)	30 (60.0%)	370 (51.9%)
- As the patient does not consult for a fall risk problem, I do not assess his risk, or his fall risk factors	50 (19.9%)	91 (22.1%)	11 (22.0%)	152 (21.3%)
- I do not manage this type of pathology in my daily practice	49 (19.5%)	78 (18.9%)	6 (12.0%)	133 (18.7%)
**Screening frequency**				
- Never	79 (26.4%)	126 (27.2%)	16 (28.6%)	221 (27.0%)
- Sometimes (for 1% to 49% of patients aged 65 and over)	99 (33.1%)	188 (40.5%)	23 (41.1%)	310 (37.9%)
- Often (for 50% to 94% of patients aged 65 and over)	90 (30.1%)	121 (26.1%)	14 (25.0%)	225 (27.5%)
- Always (for 95% to 100% of patients aged 65 and over)	31 (10.4%)	29 (6.2%)	3 (5.4%)	63 (7.7%)
**Screening responsibility by profession**				
Practitioner				
- N	299	463	57	819
- Median (IQR)	9.00 (8.00, 10.00)	8.00 (6.00, 10.00)	8.00 (7.00, 10.00)	8.00 (7.00, 10.00)
Physiotherapist				
- N	306	472	57	835
- Median (IQR)	10.00 (9.00, 10.00)	10.00 (9.00, 10.00)	10.00 (9.00, 10.00)	10.00 (9.00, 10.00)
Nursing staff				
- N	304	457	55	816
- Median (IQR)	8.00 (7.00, 10.00)	8.00 (6.00, 10.00)	8.00 (6.50, 10.00)	8.00 (6.00, 10.00)
Occupational therapist				
- N	300	448	52	800
- Median (IQR)	9.00 (8.00, 10.00)	8.00 (5.00, 10.00)	8.00 (6.00, 10.00)	8.00 (6.00, 10.00)
**Re-evaluation of fall risk**				
- No	27 (14.6%)	43 (14.1%)	5 (14.3%)	75 (14.3%)
- Yes, every patient	61 (33.0%)	76 (24.9%)	4 (11.4%)	141 (26.9%)
- Only those diagnosed as at risk of falling	97 (52.4%)	186 (61.0%)	26 (74.3%)	309 (58.9%)
**Frequency re-evaluation per year**				
- N	154	257	27	438
- Median (IQR)	6.00 (2.00, 12.00)	4.00 (0.00, 10.00)	3.00 (2.00, 6.00)	4.00 (2.00, 12.00)
**Other frequencies**				
- At the beginning and end of the referral	9 (90.0%)	5 (71.4%)	0 (0.0%)	14 (77.8%)
- Daily basis	1 (10.0%)	0 (0.0%)	0 (0.0%)	1 (5.6%)
- Depending on the case	0 (0.0%)	2 (28.6%)	1 (100.0%)	3 (16.7%)
**Risk quantification**				
- I do not classify or quantify the risk of falling	36 (14.1%)	73 (17.3%)	12 (23.5%)	121 (16.6%)
- At risk/not at risk	35 (13.7%)	91 (21.6%)	8 (15.7%)	134 (18.4%)
- No risk /low risk/moderate risk/high risk	169 (66.0%)	235 (55.8%)	26 (51.0%)	430 (59.1%)
- Probability of falling (e.g., 31%)	14 (5.5%)	17 (4.0%)	3 (5.9%)	34 (4.7%)
- Other(s)	2 (0.8%)	5 (1.2%)	2 (3.9%)	9 (1.2%)
**Systematic fall risk assessment implementation needed**				
- No	104 (41.3%)	221 (53.4%)	12 (24.0%)	337 (47.1%)
- Yes	109 (43.3%)	120 (29.0%)	22 (44.0%)	251 (35.1%)
- I do not know	26 (10.3%)	61 (14.7%)	13 (26.0%)	100 (14.0%)
- No response	13 (5.2%)	12 (2.9%)	3 (6.0%)	28 (3.9%)
*** “If a patient's fall risk is expressed as a percentage (e.g., 31%), at what level would a fall risk prevention programme be necessary?”***				
- N	243	404	46	693
- Median (IQR)	30.00 (22.50, 50.00)	30.00 (20.00, 50.00)	32.00 (24.25, 50.00)	30.00 (20.00, 50.00)

**Table 5.** Reasons for not performing a systematic fall risk assessment reported by the participants.

	French (N=79) ‡	German (N=126) ‡	Italian (N=16) ‡	Total (N=221) ‡
**Why no systematic fall risk assessment**				
- It is not my role	3 (3.8%)	10 (7.9%)	0 (0.0%)	13 (5.9%)
- I prefer to refer the patient to a specialist	9 (11.4%)	6 (4.8%)	1 (6.2%)	16 (7.2%)
- The patient is uncooperative	1 (1.3%)	3 (2.4%)	0 (0.0%)	4 (1.8%)
- I do not have sufficient technical resources	35 (44.3%)	44 (34.9%)	9 (56.2%)	88 (39.8%)
- I do not get financial recognition	14 (17.7%)	18 (14.3%)	3 (18.8%)	35 (15.8%)
- I do not have sufficient knowledge	22 (27.8%)	42 (33.3%)	3 (18.8%)	67 (30.3%)
- I do not have the time	22 (27.8%)	20 (15.9%)	7 (43.8%)	49 (22.2%)
- It is not important	3 (3.8%)	6 (4.8%)	1 (6.2%)	10 (4.5%)
- I forget to do it	9 (11.4%)	18 (14.3%)	0 (0.0%)	27 (12.2%)
- I have bad working conditions	2 (2.5%)	2 (1.6%)	1 (6.2%)	5 (2.3%)
- Other(s)	15 (19.0%)	43 (34.1%)	4 (25.0%)	62 (28.1%)

‡ Multiple responses possible, hence percentage do not add to 100%.

**Table 6.** Situations leading to fall risk assessment reported by participants.

	French (N=189) ‡	German (N=309) ‡	Italian (N=37) ‡	Total (N=535) ‡
**Situations leading to fall risk assessment**				
- If the patient says he/she fell	169 (89.4%)	267 (86.4%)	33 (89.2%)	469 (87.7%)
- If the patient says he/she is afraid of falling	166 (87.8%)	268 (86.7%)	35 (94.6%)	469 (87.7%)
- If the history (assessment) mentions one or more risk factors	137 (72.5%)	228 (73.8%)	34 (91.9%)	399 (74.6%)
- If the physiotherapy referral specifically mentions balance rehabilitation.	157 (83.1%)	244 (79.0%)	29 (78.4%)	430 (80.4%)
- If the patient consults following a trauma	109 (57.7%)	139 (45.0%)	20 (54.1%)	268 (50.1%)
- Other(s)	12 (6.3%)	12 (3.9%)	1 (2.7%)	25 (4.7%)

‡ Multiple responses possible, hence percentage do not add to 100%.

**Figure 4.**
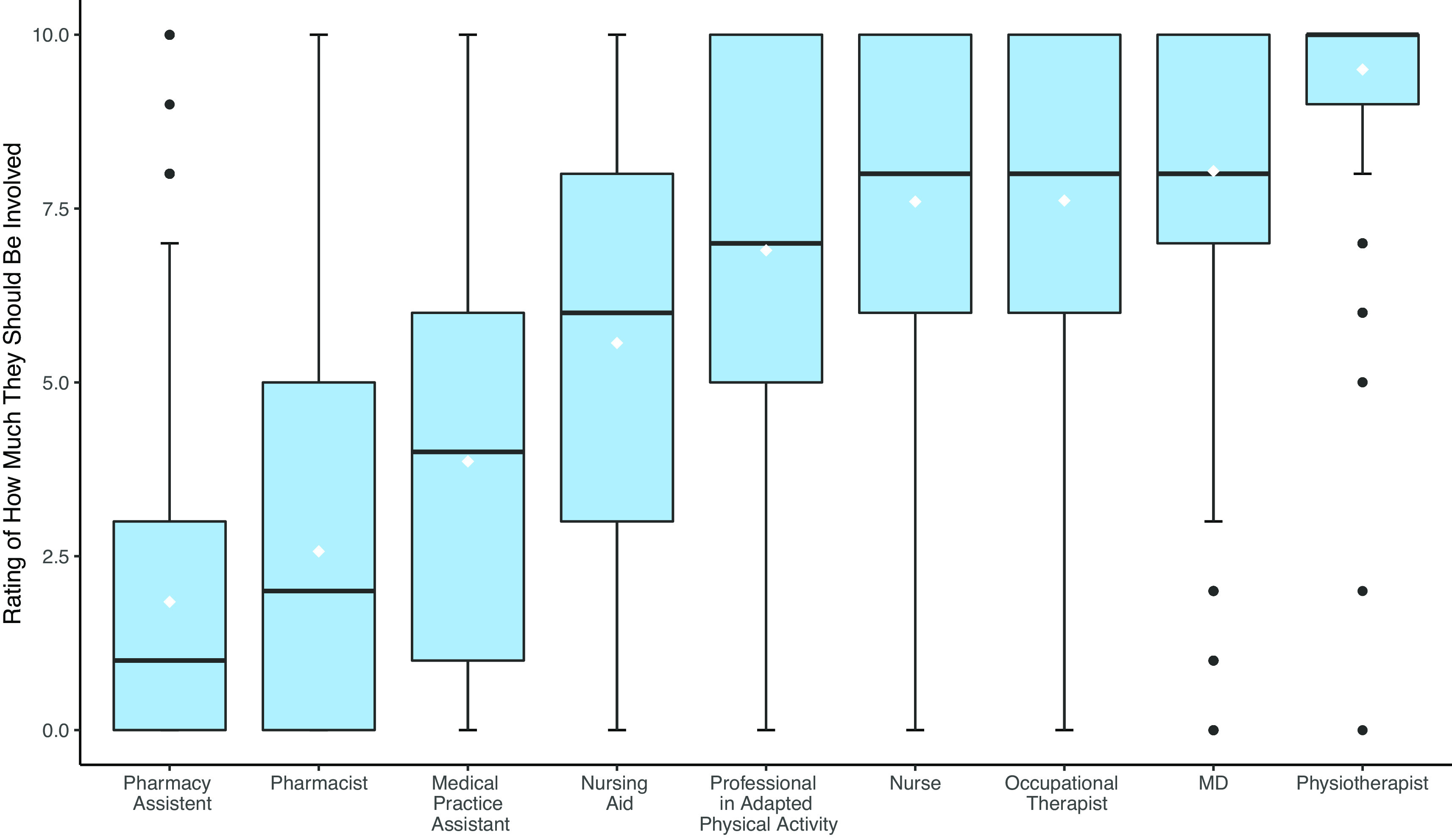
Boxplots of the rating of how much professions should be involved in fall risk assessment.

**Figure 5.**
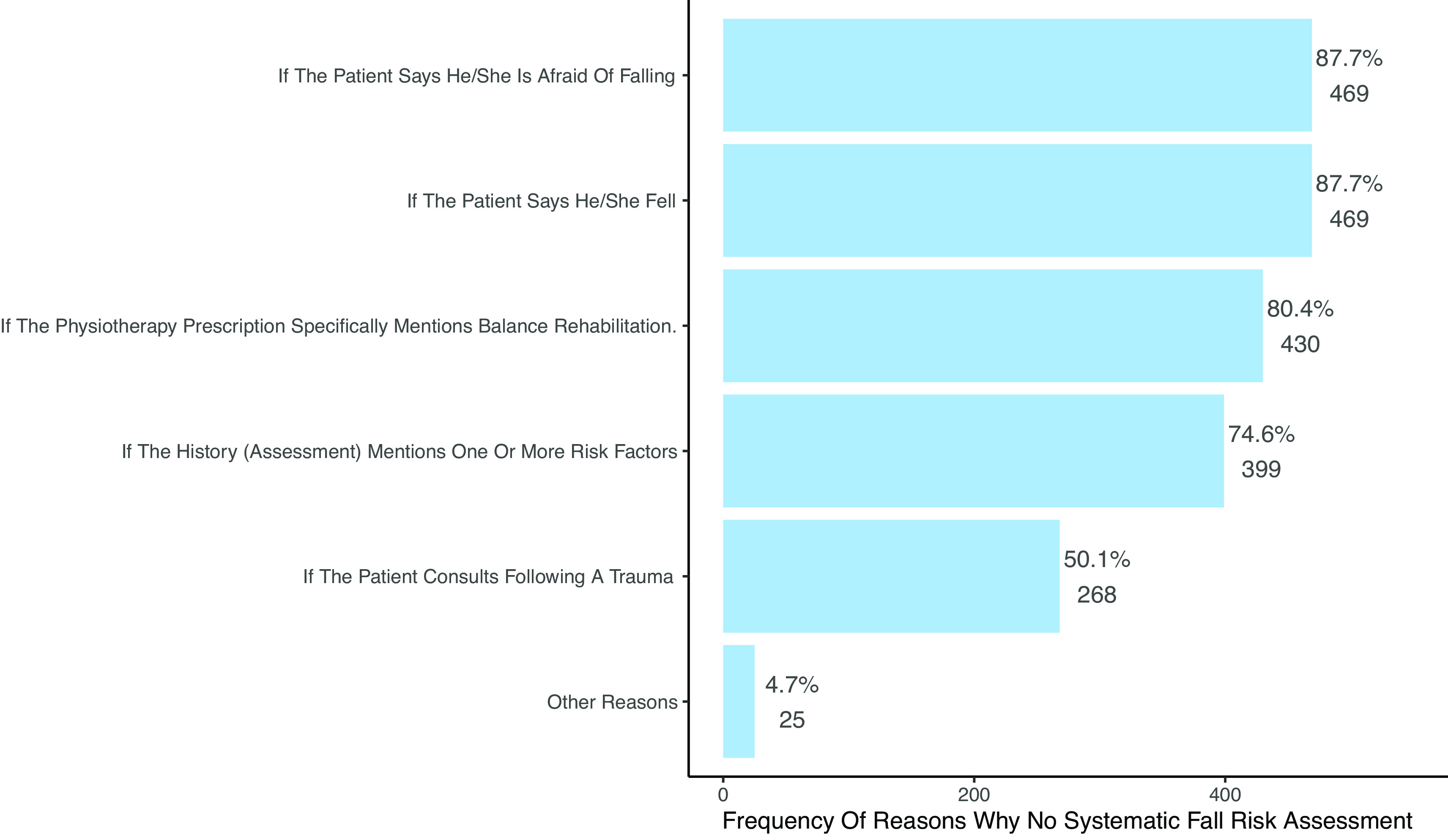
Elements that lead to screening. Multiple responses were possible, therefore percentages to not add up to 100%.

**Table 7.** Advices related to fall risk given by participants.

	Never (N=221) ‡	Sometimes (N=310) ‡	Often (N=225) ‡	Always (N=63) ‡	Total (N=819) ‡	p value
**Advice related to fall risk**						
- I generally do not give advice	5 (2.3%)	3 (1.0%)	2 (0.9%)	1 (1.6%)	11 (1.3%)	0.548
- Medication	126 (57.0%)	145 (46.8%)	106 (47.1%)	41 (65.1%)	418 (51.0%)	0.008
- Vision disorders	149 (67.4%)	235 (75.8%)	163 (72.4%)	56 (88.9%)	603 (73.6%)	0.005
- Incontinence	38 (17.2%)	51 (16.5%)	45 (20.0%)	27 (42.9%)	161 (19.7%)	<0.001
- Muscle strength	199 (90.0%)	275 (88.7%)	195 (86.7%)	60 (95.2%)	729 (89.0%)	0.258
- Joint mobility	162 (73.3%)	191 (61.6%)	147 (65.3%)	53 (84.1%)	553 (67.5%)	<0.001
- Balance (static/dynamic)	203 (91.9%)	283 (91.3%)	199 (88.4%)	62 (98.4%)	747 (91.2%)	0.096
- Walking aids	177 (80.1%)	254 (81.9%)	183 (81.3%)	59 (93.7%)	673 (82.2%)	0.092
- Home hazards	179 (81.0%)	264 (85.2%)	177 (78.7%)	58 (92.1%)	678 (82.8%)	0.042
- Inappropriate footwear	182 (82.4%)	260 (83.9%)	189 (84.0%)	58 (92.1%)	689 (84.1%)	0.319
- Malnutrition	54 (24.4%)	92 (29.7%)	66 (29.3%)	34 (54.0%)	246 (30.0%)	<0.001
- Osteoporosis and fracture risk	119 (53.8%)	168 (54.2%)	119 (52.9%)	37 (58.7%)	443 (54.1%)	0.877
- Perceived functional ability	95 (43.0%)	104 (33.5%)	82 (36.4%)	34 (54.0%)	315 (38.5%)	0.008
- Apprehension	148 (67.0%)	206 (66.5%)	155 (68.9%)	54 (85.7%)	563 (68.7%)	0.023
- Cognitive or neurological disorders	139 (62.9%)	188 (60.6%)	137 (60.9%)	46 (73.0%)	510 (62.3%)	0.300
- Cardiovascular pathology	64 (29.0%)	74 (23.9%)	59 (26.2%)	26 (41.3%)	223 (27.2%)	0.037
- Other(s)	2 (0.9%)	2 (0.6%)	0 (0.0%)	3 (4.8%)	7 (0.9%)	0.004

Sometimes = for 1% to 49% of patients over 65; Often = for 50% to 94% of patients over 65; Always = for 95% to 100% of patients over 65;

^‡^ Multiple responses possible, hence percentage do not add to 100%. P value calculated by a χ ^2^.

**Table 8.** Fall risk screening and assessment tools used by participants.

	Sometimes (N=310) ‡	Often (N=225) ‡	Always (N=63) ‡	Total (N=598) ‡	p value
**Assessments used**					
- 1 min Sit-To-Stand Test (1-mSTST)	116 (37.4%)	67 (29.8%)	20 (31.7%)	203 (33.9%)	0.170
- 30 sec Sit To Stand Test (30-STST)	115 (37.1%)	65 (28.9%)	16 (25.4%)	196 (32.8%)	0.057
- 360 Degree Turn Test (360° Turn Test)	104 (33.5%)	58 (25.8%)	19 (30.2%)	181 (30.3%)	0.155
- Aachen Falls Prevention Scale	4 (1.3%)	0 (0.0%)	1 (1.6%)	5 (0.8%)	0.213
- Activities specific Balance Confidence Scale (ABC scale)	14 (4.5%)	12 (5.3%)	5 (7.9%)	31 (5.2%)	0.532
- AGS/BGS/AAOS algorithm	0 (0.0%)	0 (0.0%)	0 (0.0%)	0 (0.0%)	
- Balance Evaluation Systems Test (BESTest)	33 (10.6%)	26 (11.6%)	7 (11.1%)	66 (11.0%)	0.946
- Balance Outcome Measure for Elder Rehabilitation (BOOMER)	4 (1.3%)	3 (1.3%)	1 (1.6%)	8 (1.3%)	0.983
- Berg Balance Scale (BBS)	166 (53.5%)	139 (61.8%)	39 (61.9%)	344 (57.5%)	0.125
- Carefall Triage Instrument (CTI)	0 (0.0%)	1 (0.4%)	0 (0.0%)	1 (0.2%)	0.436
- Clinical Test of Sensory Interaction for Balance (CTSIB)	12 (3.9%)	22 (9.8%)	9 (14.3%)	43 (7.2%)	0.002
- Community Balance and Mobility Scale (CB&M)	2 (0.6%)	3 (1.3%)	1 (1.6%)	6 (1.0%)	0.649
- Conley Scale	1 (0.3%)	0 (0.0%)	0 (0.0%)	1 (0.2%)	0.628
- Demura's Fall Risk Assessment (DFRA)	0 (0.0%)	0 (0.0%)	0 (0.0%)	0 (0.0%)	
- Downton Fall Risk Index (DFRI)	1 (0.3%)	1 (0.4%)	0 (0.0%)	2 (0.3%)	0.863
- Dynamic Gait Index (DGI)	32 (10.3%)	50 (22.2%)	15 (23.8%)	97 (16.2%)	<0.001
- Easy-Care Risk of Falls (ECRF)	1 (0.3%)	0 (0.0%)	0 (0.0%)	1 (0.2%)	0.628
- Elderly Fall Screening Test (EFST)	3 (1.0%)	5 (2.2%)	0 (0.0%)	8 (1.3%)	0.285
- Elderly Mobility Scale (EMS)	1 (0.3%)	9 (4.0%)	3 (4.8%)	13 (2.2%)	0.005
- Fall Risk Assessment and Screening Tool (FRAST)	2 (0.6%)	1 (0.4%)	0 (0.0%)	3 (0.5%)	0.794
- Fall Risk for Older People in the Community Assessment Tool (FROP-Com)	0 (0.0%)	0 (0.0%)	0 (0.0%)	0 (0.0%)	
- Fall Risk Questionnaire (FRQ)	15 (4.8%)	13 (5.8%)	2 (3.2%)	30 (5.0%)	0.690
- Fall-related Impulsive Behaviour Scale (FIBS)	3 (1.0%)	1 (0.4%)	0 (0.0%)	4 (0.7%)	0.603
- Fall Assessment Risk and Management Tool (FARAM)	1 (0.3%)	2 (0.9%)	0 (0.0%)	3 (0.5%)	0.551
- Fall Efficacy Scale International (FES-I)	12 (3.9%)	20 (8.9%)	13 (20.6%)	45 (7.5%)	<0.001
- Fall Risk Assessment Score for the Elderly (FRASE)	2 (0.6%)	1 (0.4%)	1 (1.6%)	4 (0.7%)	0.615
- Fall Risk Assessment Tool (FRAT-up)	2 (0.6%)	1 (0.4%)	0 (0.0%)	3 (0.5%)	0.794
- Fall Risk Awareness Questionnaire (FRAQ)	1 (0.3%)	4 (1.8%)	0 (0.0%)	5 (0.8%)	0.141
- Fall Risk for Hospitalised Older People Tool (FRHOP)	0 (0.0%)	0 (0.0%)	0 (0.0%)	0 (0.0%)	
- Five Time Sit to Stand Test (FTSST)	48 (15.5%)	49 (21.8%)	14 (22.2%)	111 (18.6%)	0.133
- Floor Transfer Test (FTT)	20 (6.5%)	12 (5.3%)	2 (3.2%)	34 (5.7%)	0.568
- Four Step Square Test (FSST)	10 (3.2%)	17 (7.6%)	4 (6.3%)	31 (5.2%)	0.076
- Fullerton Advanced Balance Scale (FAB)	0 (0.0%)	1 (0.4%)	0 (0.0%)	1 (0.2%)	0.436
- Functional Gait Assessment (FGA)	19 (6.1%)	26 (11.6%)	15 (23.8%)	60 (10.0%)	<0.001
- Functional Reach Test (FRT)	28 (9.0%)	38 (16.9%)	11 (17.5%)	77 (12.9%)	0.014
- Geriatric Postal Screening Survey (GPSS)	3 (1.0%)	0 (0.0%)	2 (3.2%)	5 (0.8%)	0.047
- Hendrich II Fall Risk Model	0 (0.0%)	0 (0.0%)	0 (0.0%)	0 (0.0%)	
- Hester Davis Scale (HDS)	1 (0.3%)	0 (0.0%)	0 (0.0%)	1 (0.2%)	0.628
- History of Falls Questionnaire	13 (4.2%)	10 (4.4%)	0 (0.0%)	23 (3.8%)	0.242
- Home Falls and Accidents Screening Tool (HOME FAST)	2 (0.6%)	2 (0.9%)	0 (0.0%)	4 (0.7%)	0.744
- Johns Hopkins Fall Risk Assessment Tool (JHFRAT)	0 (0.0%)	1 (0.4%)	0 (0.0%)	1 (0.2%)	0.436
- LASA fall-risk profile	0 (0.0%)	1 (0.4%)	0 (0.0%)	1 (0.2%)	0.436
- LUCAS fall risk screening	2 (0.6%)	0 (0.0%)	0 (0.0%)	2 (0.3%)	0.394
- Maximal Step Length Test (MSLT)	10 (3.2%)	5 (2.2%)	2 (3.2%)	17 (2.8%)	0.777
- Melbourne Fall Risk Assessment Tool (MFRAT)	0 (0.0%)	0 (0.0%)	0 (0.0%)	0 (0.0%)	
- Mini Balance Evaluation Systems Test (Mini BESTest)	12 (3.9%)	31 (13.8%)	9 (14.3%)	52 (8.7%)	<0.001
- Mobility Interaction Fall Chart (MIF)	0 (0.0%)	1 (0.4%)	1 (1.6%)	2 (0.3%)	0.129
- Modified Fall Assessment Tool (MFAT)	0 (0.0%)	0 (0.0%)	0 (0.0%)	0 (0.0%)	
- Modified Functional Reach Test (mFRT)	2 (0.6%)	9 (4.0%)	3 (4.8%)	14 (2.3%)	0.016
- Modified Johns Hopkins-fall risk assessment tool (mJHFRAT)	0 (0.0%)	1 (0.4%)	0 (0.0%)	1 (0.2%)	0.436
- Modified Timed-Up and Go Test (mTUG)	21 (6.8%)	35 (15.6%)	15 (23.8%)	71 (11.9%)	<0.001
- Morse Fall Scale (MFS)	0 (0.0%)	0 (0.0%)	2 (3.2%)	2 (0.3%)	<0.001
- Multifactorial Assessment Tools (MAT)	0 (0.0%)	2 (0.9%)	0 (0.0%)	2 (0.3%)	0.190
- One-Legged stance Test (OLST) /Single Leg Stance (SLS)	70 (22.6%)	54 (24.0%)	21 (33.3%)	145 (24.2%)	0.191
- Peninsula Health Fall Risk Assessment Tool (PHFRAT)	0 (0.0%)	0 (0.0%)	0 (0.0%)	0 (0.0%)	
- Physical Performance Test (PPT)	10 (3.2%)	2 (0.9%)	1 (1.6%)	13 (2.2%)	0.177
- Push and Release Test (P&R)	22 (7.1%)	22 (9.8%)	7 (11.1%)	51 (8.5%)	0.406
- Queensland Fall Risk Assessment Tool (QFRAT)	1 (0.3%)	0 (0.0%)	0 (0.0%)	1 (0.2%)	0.628
- Revised Fear of Falling Questionnaire (rFFQ)	2 (0.6%)	0 (0.0%)	0 (0.0%)	2 (0.3%)	0.394
- Self-Awareness of Fall Risk Measure (SAFRM)	4 (1.3%)	4 (1.8%)	2 (3.2%)	10 (1.7%)	0.561
- Sensory Organization Test (SOT)	5 (1.6%)	2 (0.9%)	2 (3.2%)	9 (1.5%)	0.410
- Short Falls Efficacy Scale International (Short FES-I)	0 (0.0%)	3 (1.3%)	5 (7.9%)	8 (1.3%)	<0.001
- Short Form Berg Balance Scale (SF BBS)	12 (3.9%)	14 (6.2%)	1 (1.6%)	27 (4.5%)	0.215
- Short Physical Performance Battery (SPPB)	5 (1.6%)	15 (6.7%)	5 (7.9%)	25 (4.2%)	0.005
- St Thomas Risk Assessment Tool (STRATIFY)	1 (0.3%)	0 (0.0%)	0 (0.0%)	1 (0.2%)	0.628
- Step Test	51 (16.5%)	32 (14.2%)	5 (7.9%)	88 (14.7%)	0.213
- Stopping Elderly Accident, Death and Injuries algorithm (STEADI)	1 (0.3%)	0 (0.0%)	0 (0.0%)	1 (0.2%)	0.628
- Stops Walking When Talking (SWWT)	19 (6.1%)	21 (9.3%)	9 (14.3%)	49 (8.2%)	0.072
- The Stay Independent Brochure (SIB)	3 (1.0%)	1 (0.4%)	0 (0.0%)	4 (0.7%)	0.603
- Timed-Up and Go (TUG)	136 (43.9%)	158 (70.2%)	44 (69.8%)	338 (56.5%)	<0.001
- Tinetti Balance Assessment Tool (Tinetti/POMA)	119 (38.4%)	115 (51.1%)	45 (71.4%)	279 (46.7%)	<0.001
- Walking Tests (walking speed/distance)	78 (25.2%)	68 (30.2%)	15 (23.8%)	161 (26.9%)	0.360
- Walking While Talking Test (WWT)	29 (9.4%)	19 (8.4%)	10 (15.9%)	58 (9.7%)	0.203
- Zur Balance scale (ZBS)	4 (1.3%)	0 (0.0%)	0 (0.0%)	4 (0.7%)	0.154
- Force plate	5 (1.6%)	10 (4.4%)	3 (4.8%)	18 (3.0%)	0.115
- Inertial sensor	0 (0.0%)	3 (1.3%)	0 (0.0%)	3 (0.5%)	0.082
- Sensitive walkway	7 (2.3%)	9 (4.0%)	2 (3.2%)	18 (3.0%)	0.506
- Other(s)	10 (3.2%)	12 (5.3%)	5 (7.9%)	27 (4.5%)	0.196

Sometimes = for 1% to 49% of patients over 65; Often = for 50% to 94% of patients over 65; Always = for 95% to 100% of patients over 65;

^‡^ Multiple responses possible, hence percentage do not add to 100%. P value calculated by a χ ^2^.

**Figure 6.**
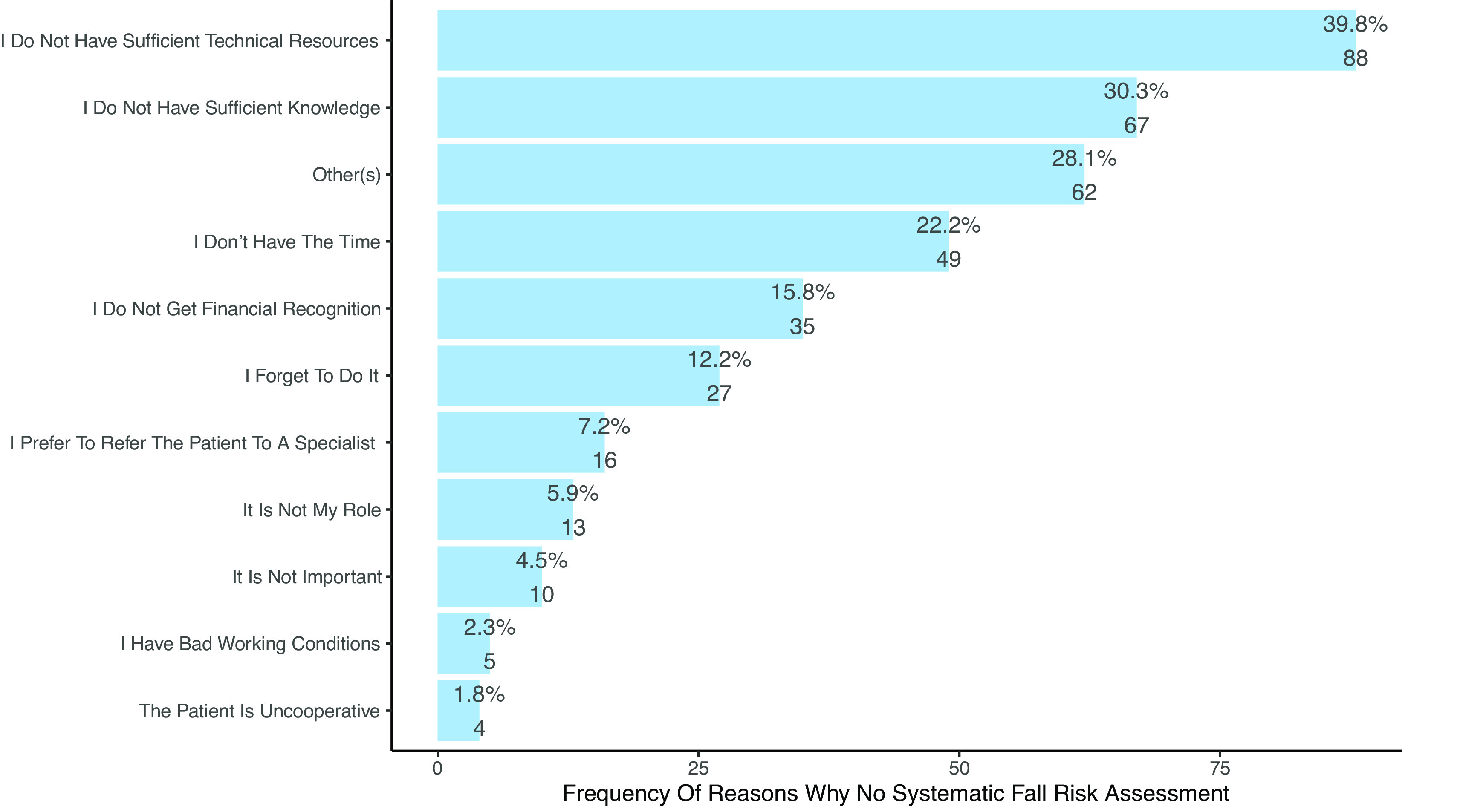
Frequency of reasons not to systematic assess fall risk. Multiple responses were possible, therefore percentages to not add up to 100%.

**Table 9.** Risk factors assessed by participants.

	Never (N=221) ‡	Sometimes (N=310) ‡	Often (N=225) ‡	Always (N=63) ‡	Total (N=819) ‡	p value
**Risk factors assessed**						
- Static balance assessment	107 (52.2%)	210 (73.9%)	170 (84.6%)	55 (88.7%)	542 (72.1%)	<0.001
- Dynamic balance assessment	97 (47.1%)	198 (69.7%)	157 (80.1%)	55 (88.7%)	507 (67.8%)	<0.001
- Muscular strength assessment	117 (57.4%)	211 (75.1%)	160 (83.8%)	58 (93.5%)	546 (74.0%)	<0.001
- Falls history assessment	176 (84.6%)	241 (85.8%)	178 (92.7%)	60 (98.4%)	655 (88.3%)	0.003
- Joint mobility assessment	164 (79.6%)	220 (78.0%)	144 (75.8%)	48 (78.7%)	576 (77.9%)	0.833
- Ask for osteoporosis	157 (75.8%)	209 (74.1%)	138 (71.9%)	45 (73.8%)	549 (74.0%)	0.845
- Perceived functional capacity assessment	161 (78.2%)	224 (80.6%)	157 (82.6%)	53 (86.9%)	595 (81.0%)	0.422
- Fear of falling assessment	146 (70.9%)	191 (69.2%)	160 (84.2%)	55 (90.2%)	552 (75.3%)	<0.001
- Ask for visual impairments	130 (63.1%)	184 (65.9%)	136 (71.6%)	46 (75.4%)	496 (67.4%)	0.152
- Cognition assessment	114 (55.3%)	151 (54.3%)	115 (59.9%)	40 (65.6%)	420 (57.0%)	0.316
- Ask for incontinence	52 (25.4%)	72 (25.9%)	56 (29.8%)	31 (51.7%)	211 (28.9%)	<0.001
- Neurological impairments assessment	156 (75.4%)	207 (73.9%)	147 (77.4%)	50 (82.0%)	560 (75.9%)	0.556
- Home hazards assessment	164 (80.4%)	233 (83.8%)	162 (84.8%)	58 (95.1%)	617 (84.1%)	0.053
- Blood pressure measurement	55 (27.2%)	109 (39.1%)	83 (43.2%)	29 (48.3%)	276 (37.7%)	0.002
- Medication review	133 (65.5%)	185 (66.3%)	135 (70.3%)	42 (70.0%)	495 (67.4%)	0.704

Sometimes = for 1% to 49% of patients over 65; Often = for 50% to 94% of patients over 65; Always = for 95% to 100% of patients over 65;

^‡^ Multiple responses possible, hence percentage do not add to 100%.

**Table 10.** Interventions offered by participants if patient screened at risk.

	Sometimes (N=310) ‡	Often (N=225) ‡	Always (N=63) ‡	Total (N=598) ‡	p value
**Interventions if patient screened at risk**					
- I inform the patient of his/her condition	252 (81.3%)	190 (84.4%)	58 (92.1%)	500 (83.6%)	0.099
- I inform my patient's doctor of his/her condition	139 (44.8%)	117 (52.0%)	43 (68.3%)	299 (50.0%)	0.002
- Even if the patient is consulting for another reason, I include some fall prevention elements in the physiotherapy sessions	245 (79.0%)	186 (82.7%)	55 (87.3%)	486 (81.3%)	0.245
- Even if the patient is consulting for another reason, I give him/her, for example, fall prevention exercises to do at home	255 (82.3%)	177 (78.7%)	54 (85.7%)	486 (81.3%)	0.365
- I assess what other modifiable risk factors are present to develop my intervention strategy (what needs to be treated)	126 (40.6%)	114 (50.7%)	41 (65.1%)	281 (47.0%)	<0.001
- I treat the patient only for the initial reason for consultation	1 (0.3%)	2 (0.9%)	1 (1.6%)	4 (0.7%)	0.467
- Other(s)	0 (0.0%)	2 (0.9%)	1 (1.6%)	3 (0.5%)	0.155

Sometimes = for 1% to 49% of patients over 65; Often = for 50% to 94% of patients over 65; Always = for 95% to 100% of patients over 65;

^‡^ Multiple responses possible, hence percentage do not add to 100%.

**Figure 7.**
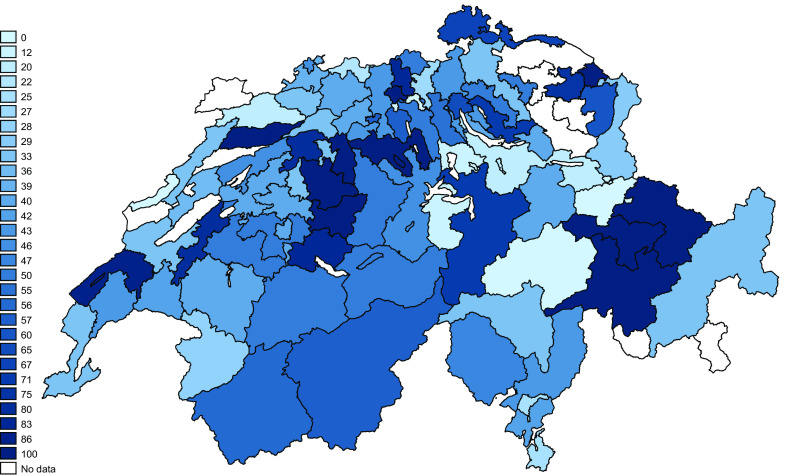
Fall risk programmes awareness according to region. The numbers in the legend correspond to the percentage of physiotherapists knowing at least one fall prevention programme (per two-digit zip region).

**Table 11.** Elements needed for systematic fall risk assessment implementation.

	Total (N=938)
**Themes**	
- Quick and easy assessment tool	181 (19.30%)
- Time	49 (5.22%)
- Checklist	38 (4.05%)
- Standardised and uniform practice	36 (3.84%)
- Training	28 (2.99%)
- List of assessments	14 (1.49%)
- Precise medical diagnosis	14 (1.49%)
- Interprofessional work	11 (1.17%)
- Financial remuneration	10 (1.07%)
- Online tool	9 (0.96%)
- Physiotherapy referrals	7 (0.75%)
- Recognition assessments value	6 (0.64%)
- History of falls	5 (0.53%)
- Clinical guidelines	4 (0.43%)
- Patient self-assessment tool	4 (0.43%)

Non mandatory open-ended question.

*Characteristics of physiotherapists assessing their patients*

First, the main characteristics related to the practice of fall risk assessment were investigated. **Hypothesis 1a (H1a):** Standardised tests or instruments are reported to be used by 580 (62%) PTs to evaluate fall risk and subjective assessment was used by 729 (77.7%) PTs (see Table 4). When participants were split into mutually exclusive categories regarding their risk assessment approach, it was observed that 49% performed a combination of subjective and standardised fall risk evaluation while 25% evaluated the risk only with a subjective assessment. Less than 5% used an instrumented evaluation (see Figure 3).

The odds of using a standardised assessment were 1.93 times higher for participants working in a clinic or hospital setting (institutional context) compared to those working in private practice (adjusted OR 1.93, 95% CI 1.37 to 2.7). **H1b:** 96 (36%) PTs working in an institutional setting and 129 (21%) of those working in private practice used a standardised instrument for 50% to 94% of their older patients. Furthermore, only 38 (14%) of those working in a clinic or hospital setting and 25 (4%) in private practice used it systematically, i.e., in at least 95% of their patients (χ ^2^ 70.8662, df 3, p <0.001). Results are presented in Table 2.

**H1c:** Among the 700 PTs treating older patients with musculoskeletal problems, 640 (91.43%) reported usually assessing them for fall risk or fall risk factors, even if they were not referred for a fall-related problem. The odds of assessing musculoskeletal patients for fall risk was 2.02 time higher for PTs engaged in an institutional setting (adjusted OR 2.02, 95% CI 1.01 to 4.04). **H1d:** Among the 666 PTs treating older patients with musculoskeletal problems, 660 (99%) reported usually assessing them for fall risk or fall risk factors, even if they were not referred for a fall-related problem. The odds of assessing neurological patients for fall risk was 1.02 time higher in PTs engaged in a clinic or hospital setting (adjusted OR 1.02, 95% CI 0.86 to 1.20). However, the 95% confidence interval range [0.86; 1.20], which was consistent with the null hypothesis that there is no difference in the fall risk assessment between PTs in institutional or private practice. **H1e:** Finally, among the 580 PTs treating older patients for respiratory diseases, 428 (74%) would usually assess the fall risk or fall risk factors in those patients even without a referral to reduce their risk of falls. PTs working in an institution did not assess these patients more often than those working in private practice (OR 1.51, 95% CI 0.98 to 2.33).

**H2:** There was a statistically significant increase in the proportion of PTs using a standardised fall risk assessment with a higher education level (test for trend, p<0.001). **H3:** Part-time PTs did not assess less than full-time PTs (OR 1.18, 95% CI 0.48 to 1.65). **H4:** PTs working alone used a standardised assessment less frequently than PTs working in a team (adjusted OR 0.52, 95% CI 0.37 to 0.72). **H5:** Finally, participants were asked to rate on a scale of 0 (not at all concerned) to 10 (very concerned) the responsibility for screening of nine health care professions. PTs practising fall risk assessment considered that this role was mainly incumbent on their profession rather than other professions (p<0.001, see Figure 4).

*Current practices in fall risk assessment*

In addition, the current practices related to the assessment of the risk of falling were considered. **H7:** The two most frequently factors leading PTs to assess their patients were the history of falls (n=469, 87.7%) and the evocation of a fear of falling (n=469, 87.7%). Receiving a specific referral to reduce the risk of falls was next (n=430, 80.4%), which only partially confirms the a priori hypothesis (see Figure 5 and Table 6).

**H9:** PTs who regularly performed fall risk assessment used a specific tool more often than those who evaluated it less often. Significant between-group differences were not always observed, but for those with p<0.05, the physiotherapist reporting evaluating fall risk “always” chose these tests more often compared to the physiotherapist assessing less often. There was one exception, the Timed-Up and Go (TUG), where therapists reporting assessing fall risk "often" (n=158, 70.2%) chose it more frequently than those who assess "always" (n=44, 69.8%). The standardised assessments most frequently employed by respondents were the Berg Balance Scale (BBS, 57.5%), the Timed-Up and Go (TUG, 56.5%), and the Tinetti Balance Assessment tool (Tinetti/POMA, 46.7%). Only a few PTs mentioned tools directly targeting fall risk such as the Stopping Elderly Accident, Death, and Injuries Algorithm (STEADI, 0.2%), the Fall Risk for Older People in the Community Assessment Tool (FROP-Com, 0%), or the AGS-BGS algorithm (0%) (see Table 8). **H10:** When assessing the risk factors of falls, PTs focused mainly on fall history (n=655, 88.3%), home hazards (n=617, 84.1%), perceived functional capacity (n=595, 81%) and joint mobility (n=576, 77.9%). All individual risk factors were assessed by at least 57% of the PTs except for blood pressure, which was assessed by 37.7% of PTs (see Table 9). **H14:** Among PTs using a standardised tool, the odds of quantifying risk was 4.24 higher than those not using it (adjusted OR 4.24, 95% CI 2.70 to 6.65). **H15:** The most common way participants did this was by classifying patient’s risk as “No risk /low risk /moderate risk/high risk” (n=430, 59.1%) or by dichotomisation “At-risk/not at risk” (n=134, 18.4%) (see Table 4). This does not support our a priori hypothesis. **H11:** Regarding the reassessment of the risk of falling, PTs who regularly assessed their patients over 65 years old also conducted re-evaluations more often than others (X ^2^ 8.9432, df 2, p = 0.0114). **H12:** In addition, PTs who systematically assessed their patients' risk of falling did 4.5 more reassessments per year than their colleagues who only “sometimes” assessed the risk (95% CI 0.33 to 8.65, adjusted regression).

*Barriers affecting systematic fall risk assessment*

**H6:** The main barriers to systematic fall risk assessment were the lack of technical resources (n=88, 39.8%), lack of theoretical knowledge (n=67, 30.3%), lack of time (n=49, 22.2%) and lack of financial recognition, i.e., reimbursement (n=35, 15.8%) (see Figure 6 and Table 5). Other barriers identified by PTs against fall risk assessment are presented in section 3.8 Content analysis (see *Barriers against fall risk assessment* and *Barriers against systematic fall risk assessment*).

*Interventions to reduce risk of falling*

PTs may undertake interventions linked with fall risk assessment. **H8:** PTs who assessed more often also provided more advice on risk factors. Indeed, whatever the risk factor for which advice was given, a higher value was observed among participants who "always" assessed their patients. However, statistically significant differences were not always observed (see Table 7). **H13:** Additionally, if a patient was found to be at risk, PTs informed them about the situation (n=500, 83.6%) and gave them exercises to do at home (n=486, 81.3%) or during physiotherapy sessions (n=486, 81.3%). In a similar situation, only a small percentage of PTs treated the patient just for the initial reason for consultation (n=4, 0.7%) (see Table 10).

*Assumptions on the implementation of guidelines recommendations*

**H16:** The opinion of the PTs regarding the implementation of a systematic assessment, i.e., of every new patient over 65 years of age, was questioned. Only 35% of the PTs agreed with this proposition (see Table 4). **H17:** A non-mandatory open-ended question was used to test this final hypothesis. The frequency and percentage of each extracted themes were calculated. Among the respondents, 181 (19.3%) desired a quick and easy-to-use tool, 49 (5.22%) more time for their initial assessments and 38 (4.05%) a checklist listing all the risk factors for falls to guide them in their assessment of patients over 65 years old. Moreover, 36 PTs (3.84%) would like to see the establishment of uniform and standardised procedures across the different health care providers in Switzerland (see Table 11).

### Geographical distribution of fall prevention programme awareness

Figure 7 shows the percentages of PTs aware of at least one programme per two-digit zip-code region. The list of known programmes mentioned, and their respective websites is presented in extended data “Fall-risk Prevention Programmes” ( Duc *et al.*, 2022).

### Exploratory analyses

A post-hoc hypothesis concerning the influence of the initial pathology on the assessment of the risk of falls was made during the statistical analysis. This result should be treated with caution.

To address the hypothesis that the assessment rate could be different in patients with musculoskeletal, neurological or respiratory pathologies, three hypothetical patient vignettes were used. In patients with musculoskeletal problems, 91% of the PTs assessed fall risk factors (99% of PTs in patients with neurological, and 74% in patients with respiratory pathologies). For the use of a standardised fall risk tool, only 11.3% of the PTs would have assessed patients with musculoskeletal problems, 48.4% of the PTs patients with neurological and 10% PTs patients with a respiratory disease). Thus, our post-hoc hypothesis that patients with neurological conditions are more frequently assessed for fall risk than those with other pathologies was confirmed.

### Content analysis 

Open-ended questions (n=13) allowed participants to express themselves freely on different aspects related to fall risk assessment. The ideas underlying some of the themes are developed below, supported by verbatim quotes in their original language. All themes extracted from the analysis of these open-ended questions are presented in extended data “Extracted Themes” ( Duc *et al.*, 2022).

**Other situations leading to fall risk assessment**

Other situations than those mentioned in the questionnaire may lead PTs to assess their patients' risk of falling. If this is not done during the initial assessment, the therapist might observe that the patient has balance problems when moving, exercising, or using walking aids. This may lead to a more detailed risk assessment. Return home after hospitalisation may also prompt PTs to assess the risk of falls of their patients.

**Other standardised assessments used**

Other assessments closely or remotely related to the risk of falls used by the PTs interviewed were: i) Functional Independence Measure (FIM), ii) Hierarchical Assessment of Balance and Mobility (HABAM), iii) Limit of stability test (LOS), iv) Modified Romberg Test (mRomberg), v) Multiple Sclerosis Questionnaire for Physiotherapists (MSQPT), vi) Romberg Test, and vii) The de Morton Mobility Index (DEMMI).

**Static balance assessment**

Static balance was assessed through various tests such as the One-Legged stance Test (OLST) or Single Leg Stance (SLS), or the 4-Stage Balance Scale Test. Some tests used such as the Berg Balance Scale (BBS) or the Balance Evaluation Systems Test (BESTest) examine both static and dynamic balance. PTs also mentioned testing balance on an unstable surface or with eyes closed without specifying whether this was done in a standardised way or not. Finally, the use of devices that also assess dynamic balance, such as the Huber 360 Evolution ^®^ or the Galileo ^®^, was also stated. Participants who did not assess static balance gave reasons such as lack of knowledge, of specific tests, or of time during the initial assessment. For others, testing did not contribute to diagnosis and management and was therefore not considered necessary. A focus was placed on the reason for the initial consultation, thus favouring treatment over assessment.

**Dynamic balance assessment**

Dynamic balance was assessed using walking tests (6 Minutes or 10 Meter Walk Tests), mixed assessments such as the Tinetti Balance Assessment Tool (Tinetti/POMA) or the Short Physical Performance Battery (SPPB), and assessments whose standardisation remains uncertain such as walking on a line, backwards, or sideways for example. Devices such as the GaitUp System ^®^ or the Dividat Senso ^®^ were also mentioned. The reasons for not assessing dynamic balance were similar to those reported for static balance. However, the lack of space to carry out the assessment was also reported.

**Muscle strength assessment**

Muscle strength was assessed by Manual Muscle Testing (according to the Medical Research Council, Kendall, Janda, Daniels), using devices such as Legpress or dynamometers or by testing some muscle groups individually such as quadriceps, triceps surae, abdominal muscles, abductors, or gluteus. Some limiting factors to muscle strength assessment were the lack of suitable assessments or devices to quantify this. Some respondents said they did not trust the 0-5 manual muscle testing accuracy, which does not assess, for example, intra-muscular coordination.

**Other risk quantification**

Another way of quantifying the risk of falls was to follow the classification proposed by the test used. However, some participants pointed out that a fixed quantification of risk was impossible. Indeed, this one could depend on the use or not of walking aids, the fluctuation of the cognitive state, of the time of day, or of the environment.

**Other advice provided in relation to the risk of falls**

Advice regarding concentration during activities of daily living (ADL), foot sensitivity in diabetes, moving around at night, sedentary lifestyle and vestibular pathologies was also provided by PTs regarding fall risk.

**Other measures undertaken if the patient is at risk**

If a patient consulted for a reason other than rehabilitation to reduce the risk of falling and the assessment revealed that they were at risk, an additional measure undertaken was to encourage the patient to discuss this directly with their general practitioner. This helps to empower the patient while allowing them freedom to determine what measures they want to take (or not take) regarding this risk.

**Barriers against fall risk assessment**

Some barriers hindered some PTs from assess fall risk of their patients aged 65 and over for the risk of falling. One of these was the lack of sense in this approach. Indeed, a well-taken anamnesis, observation of the patient during exercises, during movements in the practice (waiting room - treatment room), when undressing or dressing, during functional exercises for example, or simply experience was according to them sufficient to assess the risk of falls.

*«Through history and functional tests and inspection in the assessment, the risk is identifiable. »* (Translated from German to English by the authors.)

*«I believe that my experience is sufficient for an evaluation.”* (Translated from French to English by the authors.)

In addition, many patients were deemed not to be at risk because their gait seemed safe (observation), or they practiced a sport in their leisure time and might therefore find it questionable to assess their risk of falling. This led some therapists to focus on the initial reason for consultation mainly.

**Barriers against systematic fall risk assessment**

Barriers given against systematic assessment of patients over 65 are various. First, the age criterion alone was not sufficient to justify a systematic risk assessment according to some participants. 75-80 years old would be more appropriate for a so-called "systematic" assessment. In addition, this supplementary evaluation was perceived to be too time-consuming.

*"As I find the age limit for a systematic assessment of every patient of 65 years old too low, and we do not have the time and resources to clarify the risk of falls in all these patients regardless of the diagnoses and the A-Z. A large part of the patients I experience as still very fit and active at the age of 65."* (Translated from German to English by the authors.)

For some, a case-by-case approach would therefore be preferred in the first instance to a systematic procedure. The patient should be assessed according to his level of activity, his pathologies, his anamnesis, his antecedents, the subjective and objective assessment elements, of the general impression for example. However, other participants considered that everyone should be assessed because " *prevention is better than cure*". Patients are often referred to PTs too late when the situation is already complex (generalised deconditioning, significant and/or disabling joint damage and cognitive disorders…).

## Discussion

This online cross-sectional survey conducted in three language regions of Switzerland and including 938 participants aimed to investigate the current practices of physiotherapists in fall prevention. There were the main findings: i) 62% of physiotherapists perform a standardised fall risk assessment and most of them evaluate the different fall risk factors (e.g., balance or muscular strength). However, only 14% of physiotherapists working in an institutional context and 4% of those in a private practice carry it out in a systematic way. ii) The proportion of physiotherapists using a standardised risk assessment was higher in those with higher education compared to those with lower education, those working full time compared to those working part-time, in those working in teams compared to those working alone, and in those working in clinics or hospitals compared to those working in private practices. iii) Barriers to conducting a fall risk assessment were commonly a lack of technical resources, lack of theoretical knowledge, lack of time and lack of reimbursement. iv) Only 35% of the physiotherapists think that a systematic fall risk assessment of every new patient over 65 years of age should be implemented. v) The most frequent elements that would facilitate a more systematic fall risk assessment are the use of a quick and easy-to-use assessment tool, more time for the assessments and a checklist listing all the risk factors for falls to guide physiotherapists. Moreover, physiotherapists would like to see the establishment of uniform standardised procedures between the different health care providers in Switzerland.

**Current practices in assessing the risk of falls in patients over 65 years old**

Only 14% of physiotherapists working in an institutional context and 4% of those in a private practice used a standardised test or instrument to assess their patients' risk of falling in a systematic way (i.e., in at least 95% of their patients). Moreover, a disparity in the rate of assessment was also observable according to the initial pathologies (musculoskeletal, neurologic, respiratory). Annual assessment for the risk of falls among every patient 65 years of age or older is recommended ( American Geriatrics Society & British Geriatrics Society, 2011). In the light of our results, efforts are still needed to ensure that all patients over 65 are assessed by physiotherapists or other health care providers at least once a year. The implementation of a standardised risk assessment in physiotherapists with lower education, working in private practice, either alone or part-time, should notably be supported considering potential barriers.

The Berg Balance Scale (BBS) and the Timed-Up and Go test (TUG) were the two most frequently assessments of fall risk used. Many physiotherapists also used the One-Legged Stance Test (OLST) or Single Leg Stance (SLS). These results were similarly observed in two other studies conducted in Australia and Canada with physiotherapists working in the geriatrics field ( Ackerman *et al.*, 2019; Sibley *et al.*, 2011) suggesting that these tools are well known and implemented among physiotherapists. In their meta-analysis, Lusardi *et al.*, (2017) concluded that the BBS, the TUG and the Five Time Sit-To-Stand (FTSS) were the most evidence-supported functional measures to determine individual risk of future falls. However, a multifactorial assessment is required to evaluate the risk factors for falls ( National Institute for Health and Care Excellence, 2013). It should be underlined that we did not analyse whether participants used these tools in isolation or combination.

Generally, our results demonstrated a high level of involvement in assessing and advising on risk factors for falls. According literature, history of falls frequently showed a strong association with the risk of falls (odds ratios between 2.4 and 2.6) ( National Institute for Health and Care Excellence, 2013). In line with these recommendations, 88.3% (n=655) of physiotherapists in Switzerland assess the history of falls. Another self-reported risk factor for falls is the perceived functional capacity. Indeed, Porto *et al.* (2020) showed that a poor consideration of self-general health increases the risk of falling (adjusted OR 2.24, 95% CI 1.14 to 4.42, p-value 0.019). This evaluation seems to be also well established among participants as 81% of them (n=595) asked their patients about their perceived functional capacity.

More than 60% of physiotherapists in Switzerland evaluate their patients' medication. This means that they are aware that too many medications and combinations of medications, especially psychotropic and anti-hypertensive drugs, are a risk factor for falls ( Ang *et al.*, 2018; Hill & Wee, 2012; Park *et al.*, 2015). However, the results of our study show that the degree of involvement of pharmacists, especially pharmacy assistants, varies widely according to the participants. This may suggest that physiotherapists do not work consistently with these professionals. It is known that the medication of older people should be reviewed with a specialist and modified or discontinued if possible ( National Institute for Health and Care Excellence, 2013). In Switzerland, physiotherapists or pharmacists do not have any decision-making power regarding medication. Their common action would possibly be to contact the treating physician to see if certain medications could be removed or even replaced to avoid increasing the risk of falling. However, some collaborative projects between physiotherapists and pharmacists have been initiated in Switzerland. As part of its 100th anniversary, the Geneva Association of Physiotherapy decided to organise a campaign in collaboration with the Geneva pharmacists' association, Pharma Genève, on the theme: “Comment prévenir les chutes”. This campaign took place in the participating pharmacies from 25 September to 7 October 2017. The objective was to raise awareness of this risk that costs so much to society (and to our insurances) and above all the importance of maintaining physical activities whatever your age. Tests were carried out by pharmacy assistants, pharmacists and physiotherapists and advice on modifiable risk factors were given to the population ( Mulhauser-Wallin & Haas, 2017).

More generally, in Switzerland, the standardisation of interprofessional collaboration, although supported, still faces many barriers (financial, lack of time). This kind of approach should be developed and strengthened in Switzerland, particularly in the prevention of falls ( Baumann *et al.*, 2022).

It should also not to be forgotten that the use of screening tools can also carry certain risks. For example, if a screening test has moderate or low sensitivity and is applied in a population with a high incidence of falls. If the test is negative, there is a high risk that health care providers will mistakenly believe that the older person is not at increased risk of falls ( Oliver, 2008).

**Barriers related to fall risk assessment**

According to our results, fall risk is often not assessed with a standardised approach. Indeed, one-quarter of physiotherapists (n=235, 25%) exclusively performed a subjective fall risk assessment mainly based on observation, anamnesis, or experience. Test-retest reliability may be lower in non-standardised tests than in standardised ones, making it less easy to reassess risk. Furthermore, this may result in the omission of some risk factors, which would be contrary to the recommendations of the guidelines in this area on multifactorial risk assessment ( American Geriatrics Society & British Geriatrics Society, 2011; National Institute for Health and Care Excellence, 2013). Among the range of tools designed to standardise this procedure, the Centers for Disease Control and Prevention (CDC) developed the Stopping Elderly Accidents, Death and Injuries algorithm ( STEADI). To screen for risk of falling, this valid instrument proposes the Stay Independent Brochure, a fall risk self-assessment tool, or the Three Key Questions, *i) "Have you fallen in the last year?” ii) “Do you feel unsteady when standing or walking?” iii) “Do you worry about falling?”.* If the patient is at risk, further assessments are provided to evaluate potential risk factors and define appropriate interventions ( Nithman & Vincenzo, 2019; Stevens & Phelan, 2013).

The survey also highlighted other barriers like the lack of technical resources, theoretical knowledge, time, and financial recognition. Similar findings were made in studies evaluating the practices of fall risk assessment of patients by physiotherapists working with osteoarthritis patients (lack of time: 74%) ( Ackerman *et al.*, 2019) or GPs (lack of time: 13.3%, of knowledge: 13.3%, of financial compensation: 11.1%) ( Gaboreau *et al.*, 2016). To address those issues, assessment of potential risk could be easily implemented using the Three Key Questions for example. Their application allowed 95% of patients at high risk to be identified while potentially decreasing the screening duration ( Eckstrom *et al.*, 2017).

Finally, only 35% of physiotherapists stated that systematic fall risk assessment should be performed. The notion of systematic implies “every new patient over 65 years of age”. Several participants stated that the age of 65 was not appropriate due to the heterogeneity of patients in this age group, stating that 75-80 years would be more appropriate for a systematic procedure. However, if we look at the Swiss statistics on falls during the year 2017, we can see that the proportion of fallers increases by 10% between people aged 65-79 and those over 80 ( BFS, 2019). This demonstrates the importance of fall risk assessment at an early stage.

**Interventions to reduce risk of falling**

Identifying individual fall risk factors allows planning a specific fall prevention strategy even if the risk is mild ( Hill, 2009). In case of proven risk, 81.3% (n=486) of the respondents gave their patients exercises at home or included them during therapy. According to the literature, those should be individualised and based on the identified risks to prevent future falls ( Hill, 2009; Cheryl & Butcher, 2017), but our survey did not consider this aspect.

Regarding interventions, it is also interesting to highlight the results of a randomised controlled trial. Greenberg *et al.* (2020) found that the adoption of a decision tool, in this case the STEADI algorithm, generally encouraged subjects to take part in individualised interventions more than controls. This suggests that using a standardised tool effectively encourages patients to act on their risk of falling.

### Clinical implications

Despite the multitude of tests available, many participants would like a quick and easy-to-use assessment tool or a checklist listing all the risk factors for falls to guide them in assessing patients over 65 years old. The implementation in Switzerland of a tool directly targeting fall risk assessment such as the STEADI algorithm, could be relevant. Indeed, it was specially created to help caregivers to perform fall risk assessments and treat patients appropriately ( Stevens & Phelan, 2013). Physiotherapists working in private practice, either alone or part-time, should particularly be supported to use standardised assessments. Improvements in basic education, continuing education courses, online materials, financial incentives (i.e., remuneration for assessments), and interprofessional prevention campaigns could also potentially increase the implementation of established best practices in fall prevention.

In addition, many therapists called for the standardisation of current practices between different health care providers. This would enable the adoption of a "common language" in terms of testing, risk quantification and interventions. According to Gaboreau *et al.* 65.3% of French GPs considered the implementation of an annual falls risk assessment useful. Unfortunately, only 28.8% of them performed it each year ( Gaboreau *et al.*, 2016). To optimise practices related to falls prevention, it is therefore essential to increase the awareness and involvement of each health professional. More generally, there is still room for improvement in the quality of care, coordination between the treating physician and the specialist, and follow-up care after discharge from hospital in Switzerland ( Merçay, 2017).

### Future studies

Future studies are needed to develop a fall risk assessment adapted to the needs and constraints of physiotherapist’s practice in Switzerland. This tool could be based on the STEADI, which was proven to reduce falls-related hospital admissions in older people and associated health care costs ( Johnston *et al.*, 2019). Then, local implementation studies including in-depth qualitative interviews with sub-groups of physiotherapists to identify potential barriers and facilitators could be planned. This tool could thus serve as a basis for discussion and joint decision-making among health professionals.

### Limitations

**Participant’s recruitment**

One limitation was, that we could no address all, or a random sampling of all physiotherapists working in Switzerland. In the absence of a federal register comprising all currently practising physiotherapists in Switzerland, a recruitment strategy was developed to maximise the results generalisability and sample representativeness. The research team sent more than 3600 emails and the partner associations and schools to set up our sample. However, this strategy did not avoid the phenomenon of overlapping; obviously, some physiotherapists have been contacted in several ways while others were omitted. Moreover, physiotherapists who took part may have been more sensitive to this topic than non-respondents, potentially influencing the results. The generalisation of cross-sectional survey findings is often discussed in the literature. For some authors, sources of disparity within the population studied do not allow the results to be generalised between the individuals contacted and the others ( Andrews *et al.*, 2003). For others, due to the large number of individuals contacted, this design is more likely than others to acquire data from a representative sample and thus allow extrapolation ( Kelley *et al.*, 2003). We assumed that our sample is representative of all physiotherapists working in Switzerland but are not able to test this and therefore these findings should be interpreted with caution. Nonetheless, this study provides a snapshot of the current global situation in Switzerland regarding fall risk assessment, considering that the physiotherapist's practices and knowledge are in constant evolution.

A further limitation was, that few questions showed low reliability. However, these were multiple choice items with many response options. For example, the question that targeted different fall risk assessment tools had 81 different response options.

**Open-ended questions analysis**

A quantitative design was chosen to answer questions based on the current practices of physiotherapists in Switzerland in fall prevention. Without pretending to fulfil the criteria of a mixed design, but to allow participants to specify or clarify their answers, twelve open-ended questions were also asked. The analysis of the participants' answers was limited to a content analysis based on the inductive approach proposed by Elo and Kyngäs and Graneheim and Lundman ( Elo & Kyngäs, 2008; Graneheim & Lundman, 2004). The lead author (MD) was responsible for summarising each response and then classifying them according to general recurring themes. These were then counted to determine their frequency. This work, although long and tedious, was fascinating. It raised relevant aspects concerning the needs of physiotherapists and the future reflections to be carried out to prevent falls in our country. However, it would have been relevant to carry out this work with the help of a third person to ensure that the answers were well understood and that the classification by themes was adequate and unanimous.

### Strengths

First, to our knowledge, it is the first study to explore this topic in Switzerland. Fall prevention is of high importance to improve the care of our older patients. Guidelines have underlined the effectiveness of multifactorial assessment to determine an individual’s risk of falling and implement suitable prevention strategies ( American Geriatrics Society & British Geriatrics Society, 2011; Beauchet *et al.*, 2011; Feder *et al.*, 2000; Moreland *et al.*, 2003; National Institute for Health and Care Excellence, 2013). It was, therefore, necessary to review current practices and to propose measures according to physiotherapists needs. Furthermore, our recruitment strategy and the development of a questionnaire in three languages (French, German, Italian) enabled nationwide participation, with the final sample size exceeding our initial expectations. In addition, this study led some participants to reflect on their current practices.

## Conclusions

In conclusion, this study showed that most physiotherapists working in Switzerland perform some form of fall risk assessment. Moreover, many of them are aware of the problem as they frequently assess and advise on risk factors for falls to their patients over 65 years old. However, despite current recommendations, risk assessment itself is still too often unsystematic and based on subjective criteria. Measures are required to foster the use of standardised assessments by mitigating existing barriers and increasing incentives. To overcome this challenge, education plays an important role and should emphasise the importance of objective fall risk assessments with the use of appropriate screening tools. In addition, the development of an assessment to facilitate the implementation of a fall prevention strategy based on best practice should be considered. This would improve adequate care for our older adults while relieving the health system of the costs associated with this issue.

## Data availability

### Underlying data

Open Science Framework: Current Practices of Physiotherapists in Switzerland Regarding Fall Risk-Assessment for Community-Dwelling Older Adults: A National Cross-Sectional Survey, https://doi.org/10.17605/OSF.IO/MP9U4 ( Duc *et al.*, 2022).

This project contains the following underlying data:

- df_csv.csv

### Extended data

Open Science Framework: Current Practices of Physiotherapists in Switzerland Regarding Fall Risk-Assessment for Community-Dwelling Older Adults: A National Cross-Sectional Survey, https://doi.org/10.17605/OSF.IO/MP9U4 ( Duc *et al.*, 2022).

This project contains the following extended data:

− CHERRIES Checklist.pdf
− Designing Tools.pdf
− Survey.pdf
− Survey Development.pdf
− Survey Pre-testing and Validation.pdf
− Comprehensibility Assessment.pdf
− A-priori Hypotheses.pdf
− Survey Send Out.pdf
− Cover Letter.pdf
− Missing Values.pdf
− Fall-risk Prevention Programmes.pdf
− Extracted Themes.pdf

Data are available under the terms of the Creative Commons Attribution 4.0 International license (CC-BY 4.0).
