# Supplementary material for: Current practices of physiotherapists in Switzerland regarding fall risk-assessment for community-dwelling older adults: A national cross-sectional survey
Source: F1000Res. 2023 Dec 11;11:513. Originally published 2022 May 12. [Version 2] doi: 10.12688/f1000research.73636.2 (PMC10733665; doi:10.12688/f1000research.73636.2)
Supplement: Peer review history

# Reviewer report: Reviewer response for version 2

**DOI:** 10.5256/f1000research.159032.r229093

**Published:** 2023-12-20

**Authors:** Arthur de Sá Ferreira

**Affiliations:** 1 Postgraduate Program in Rehabilitation Sciences, Centro Universitário Augusto Motta, Rio de Janeiro, RJ, Brazil

Thank you for the opportunity to discuss your manuscript. All comments were adequately addressed. I have no new comments.

Is the work clearly and accurately presented and does it cite the current literature?

Yes

If applicable, is the statistical analysis and its interpretation appropriate?

Yes

Are all the source data underlying the results available to ensure full reproducibility?

Yes

Is the study design appropriate and is the work technically sound?

Yes

Are the conclusions drawn adequately supported by the results?

Yes

Are sufficient details of methods and analysis provided to allow replication by others?

Yes

Reviewer Expertise:

Rehabilitation, Physical Therapy, Biomedical Engineering, Biostatistics

I confirm that I have read this submission and believe that I have an appropriate level of expertise to confirm that it is of an acceptable scientific standard.

# Reviewer report: Reviewer response for version 1

**DOI:** 10.5256/f1000research.77300.r180009

**Published:** 2023-08-24

**Authors:** Tobias Braun

**Affiliations:** 1, Hochschule für Gesundheit, Bochum, Germany

Thank you for the opportunity to review this manuscript. I have read it with great interest.

 The authors performed a Swiss-wide national cross-sectional survey on the current practices of physiotherapists regarding fall risk-assessment for community-dwelling older adults.

 I have reviewed the manuscript and would like to make some minor suggestions on how to improve the quality of reporting. This manuscript is well-written and completely reported, well-structured and easy to read/follow. I gratulate the authors for their great work, which will inform the health care of older people at a high risk of falling.

 Title & Abstract:

1. Well reported. Minor comment: decimal places for relative figures should be reported consistently (either one or no decimal place, please).

Introduction/Background:

1. I recommend avoiding the term “elderly” and use “older adults” etc. instead.
2. I terms/words such as “mainly” are used, please provide exact figures (e.g., “… are mainly attributable to falls” – how many?).
3. I recommend referring also to the currently published world guidelines for falls prevention and management for older adults ^1^.

Methods:

1. The methods are reported very transparently, completely and structured logically. Reporting according to CHERRIES has been performed very thoroughly.

 

 Results:

1. The flow chart (fig 1) is a bit confusing at the lower part. It seems that the bloc “eligible physiotherapists (n=938)” separate into 3 blocs (participants “flow” into the three blocs) and that the numbers of the three blocs add up to 938. However, it actually separates into 2 blocs (714 + 224; left and right), and the “middle bloc” seems to be the “sum” of the other 2 blocs, and might be placed at the “bottom”. Otherwise, the “flow” of participants seems not correct according to the numbers.
2. I recommend reporting relative numbers in relation to the total sample size consistently, with one decimal place, e.g. “224 (23.88%)” -- > 224/938 (23.9%).
3. I was a bit confused by some headings of the tables, e.g., “Table 4. Hypotheses 1, 2, 3, 4, 5, 11, 12, 14, 15, 16.” or “Table 5. Hypothesis 6.”. The authors might please consider to use more explicit titles, if possible, e.g., “Table 5. Reasons for not performing a systematic fall risk assessment reported by the participants”, and so on.

Discussion:

1. Well reported, no comments.

Is the work clearly and accurately presented and does it cite the current literature?

Yes

If applicable, is the statistical analysis and its interpretation appropriate?

Yes

Are all the source data underlying the results available to ensure full reproducibility?

Yes

Is the study design appropriate and is the work technically sound?

Yes

Are the conclusions drawn adequately supported by the results?

Yes

Are sufficient details of methods and analysis provided to allow replication by others?

Yes

Reviewer Expertise:

physiotherapy, rehabilitation, older adults, neurorehabilitation, fall risk, evidence-based practice

I confirm that I have read this submission and believe that I have an appropriate level of expertise to confirm that it is of an acceptable scientific standard.

## References

[1] : World guidelines for falls prevention and management for older adults: a global initiative. *Age Ageing* .2022;51(9) : 10.1093/ageing/afac205 10.1093/ageing/afac205 36178003 PMC9523684

# Reviewer report: Reviewer response for version 1

**DOI:** 10.5256/f1000research.77300.r150913

**Published:** 2022-09-21

**Authors:** Arthur de Sá Ferreira

**Affiliations:** 1 Postgraduate Program in Rehabilitation Sciences, Centro Universitário Augusto Motta, Rio de Janeiro, RJ, Brazil

Manuscript title: Current practices of physiotherapists in Switzerland regarding fall risk-assessment for community-dwelling older adults: A national cross-sectional survey

 Comments:

 This manuscript reports an online survey study designed to evaluate to what extent physiotherapists carry out fall risk assessments as the first step toward a fall prevention intervention in the population of community-dwelling older adults in Switzerland. The manuscript is written following related guidelines and checklists (e.g., CHERRIES). The background and rationale of the study are clearly posed, with objective aims. The study design seems adequate to the study aims. Given the sampling scheme, the researchers implemented strategies to increase nationwide coverage of the survey and responses. The procedures for development, validity/reliability assessment, and implementation of the questionnaires are fully described and seem sufficient to allow replication. Data analysis follows current best practices labeling the a priori and exploratory hypotheses. Results are very well organized and clearly linked both to the methods and hypotheses. I commend the authors for the well-conducted and reported study. I have only a few comments I would like the authors to consider.

 Minor comments:

1. Results, reliability. Consider double-checking whether the observed kappa values were paradoxically low. Other reliability coefficients can be used in such cases ^1^.
2. It is curious that polypharmacy, mentioned in the Introduction section, is not presented in Results nor further discussed. The large variability for rating how much the pharmacy assistant/pharmacist should be involved (Figure 4) is intriguing. It may suggest that most PTs do not regard polypharmacy as a criterion for a high risk of falling. Conversely, it may also suggest that PTs who acknowledge the whole of polypharmacy in risk stratification score higher for enrolling pharmacy assistances/pharmacists. I would like to know the authors’ opinion on this matter.

Is the work clearly and accurately presented and does it cite the current literature?

Yes

If applicable, is the statistical analysis and its interpretation appropriate?

Yes

Are all the source data underlying the results available to ensure full reproducibility?

Yes

Is the study design appropriate and is the work technically sound?

Yes

Are the conclusions drawn adequately supported by the results?

Yes

Are sufficient details of methods and analysis provided to allow replication by others?

Yes

Reviewer Expertise:

Rehabilitation, Physical Therapy, Biomedical Engineering, Biostatistics

I confirm that I have read this submission and believe that I have an appropriate level of expertise to confirm that it is of an acceptable scientific standard, however I have significant reservations, as outlined above.

## References

[1] : Computing inter-rater reliability and its variance in the presence of high agreement. *Br J Math Stat Psychol* .2008;61(Pt 1) : 10.1348/000711006X126600 29-48 10.1348/000711006X126600 18482474

# Response

**Published:** 2023-11-16

**Authors:** Morgane Duc

**Affiliations:** University of Applied Sciences of Western Switzerland, Switzerland

Hello, thank you for your very pertinent feedback.

 Regarding the first point, we now calculated Prevalence and Bias Adjusted kappa (PABAK, also called Brenner-Prediger Kappa) and the values were not substantially higher, therefore we prefer to leave the original kappa calculations.

 Suggestions regarding your second point will be made in the final document. 

 Best regards 

 Morgane Duc 

 Roger Hilfiker
